# Development of amplicon sequencing for the analysis of benzimidazole resistance allele frequencies in field populations of gastrointestinal nematodes

**DOI:** 10.1016/j.ijpddr.2019.08.003

**Published:** 2019-08-13

**Authors:** Neil D. Sargison, Madison MacLeay, Alison A. Morrison, David J. Bartley, Mike Evans, Umer Chaudhry

**Affiliations:** a University of Edinburgh Roslin Institute and Royal (Dick) School of Veterinary Studies, Easter Bush Veterinary Centre, Roslin, Midlothian, EH25 9RG, United Kingdom; b Moredun Research Institute, Pentlands Science Park, Bush Loan, Penicuik, EH26 0PZ, United Kingdom

**Keywords:** Gastrointestinal nematode, *Teladorsagia circumcincta*, Anthelmintic, Benzimidazole, Isotype 1 β-tubulin locus, Deep amplicon sequencing

## Abstract

Anthelmintic resistant gastrointestinal helminths have become a major cause of poor health in sheep and goats. Sensitive and specific molecular markers are needed to monitor the genotypic frequency of resistance in field parasite populations. Gastrointestinal nematode resistance to benzimidazole is caused by a mutation in one of three positions within the isotype 1 β-tubulin gene. In the absence of markers for resistance to the other broad spectrum anthelmintic classes, these provide a relevant study example. Determination of the prevalence of these single nucleotide polymorphisms in field nematode populations can be impractical using conventional molecular methods to examine individual parasites; which can be laborious and lack sensitivity in determining low levels of resistance in parasite populations. Here, we report the development of a novel method based on an Illumina MiSeq deep amplicon sequencing platform to sequence the isotype 1 β-tubulin locus of the small ruminant gastrointestinal nematode, *Teladorsagia circumcincta*, and determine the frequency of the benzimidazole resistance mutations. We validated the method by assessing sequence representation bias, comparing the results of Illumina MiSeq and pyrosequencing, and applying the method to populations containing known proportions of resistant and susceptible larvae. We applied the method to field samples collected from ewes and lambs on over a period of one year on three farms, each highlighting different aspects of sheep management and approaches to parasite control. The results show opportunities to build hypotheses with reference to selection pressures leading to differences in resistance allele frequencies between sampling dates, farms and ewes or lambs, and to consider the impact of their genetic fixation or otherwise. This study provides proof of concept of a practical, accurate, sensitive and scalable method to determine frequency of anthelmintic resistance mutations in gastrointestinal nematodes in field studies and as a management tool for livestock farmers.

## Highlights

• Development and validation of Illumina Mi-Seq for the detection of anthelmintic resistance.
• Assessment of the novel assay using benzimidazole resistance as a proof of concept.
• Novel approach open the area of research to study the anthelmintic resistance.

## Graphical abstract

**Figure:**
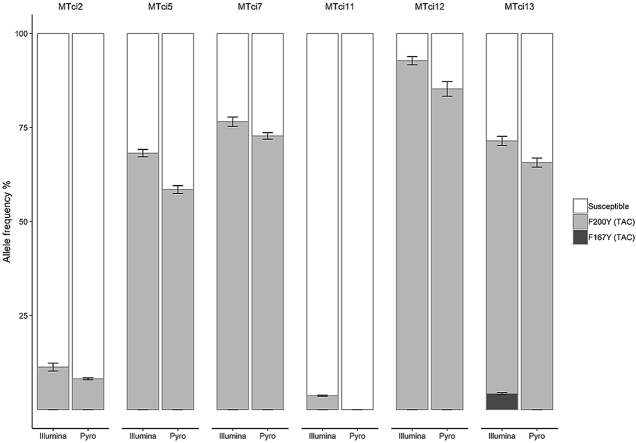

## 1 Introduction

Resistance to broad spectrum anthelmintic drugs is a global threat to efficient livestock production (Kaplan, 2004; Kaplan and Vidyashankar, 2012). Anthelmintic drugs belonging to the tubulin binding benzimidazole class are the mainstay for the control gastrointestinal nematode in lower and middle income countries (Ali et al., 2019; Chaudhry et al., 2015). The benzimidazole drugs are also routinely used in intensive small ruminant production throughout Europe, despite the widespread emergence and gene flow of resistance to the drug group in most small ruminant production limiting gastrointestinal nematode species (Borgsteede et al., 1991; Fleming et al., 2006). A large-scale survey of UK sheep farms showed the benzimidazole resistance phenotype in 64–77% of farms, almost exclusively in the predominant gastrointestinal nematode species, *Teladorsagia circumcincta* (Bartley et al., 2003; Mitchell et al., 2010). Benzimidazole resistance is an emerging problem around the world in regions where efficient small ruminant production is essential in poverty alleviation (Lalljee et al., 2018) and where alternative anthelmintic drug groups are hitherto unavailable or prohibitively expensive (Sargison et al., 2017; Van Wyk et al., 1989; Wong and Sargison, 2018).

Benzimidazole resistance is associated with mutations in the isotype 1 β-tubulin gene that prevent drug binding (Geary et al., 1992a). A single nucleotide polymorphism (SNP) mutation was first identified in benzimidazole resistant *Haemonchus contortus* and *Trichostrongylus colubriformis* resulting in an amino acid substitution from tyrosine to phenylalanine at position 200 (F200Y) of the polypeptide encoded by the isotype 1 β-tubulin gene (Kwa et al., 1994, 1995). The same point mutation was also shown in benzimidazole resistant *T. circumcincta* (Elard et al., 1996). This F200Y SNP is considered to be the most important mutation conferring benzimidazole resistance, and has been shown to be functionally significant with respect to the benzimidazole resistance phenotype by transfection of the gene into *Caenorhabditis elegans* (Kwa et al., 1995). A substitution from tyrosine to phenylalanine at position 167 (F167Y) of the polypeptide encoded by the isotype 1 β-tubulin gene has been found in *T. circumcincta* and *H. contortus* (Silvestre and Cabaret, 2002). A third isotype 1 β-tubulin mutation (E198A) with substitution from glutamate to alanine at position 198 was first identified in field populations of *H. contortus* (Chaudhry et al., 2015; Ghisi et al., 2007; Rufener et al., 2009) and has subsequently been reported in *T. circumcincta* with two substitutions (E198L [TTA] or E198A [GCA]) (Redman et al., 2015).

The emergence and spread of benzimidazole resistance in parasite populations is aided by high effective population sizes and high mutation rates (Redman et al., 2015). Selection for anthelmintic resistance is influenced by the timing and frequency of treatments with reference to the proportion of the total gastrointestinal nematode population that is exposed to the drug, or in a refuge from exposure, referred to as in refugia (van Wyk, 2001). Pragmatic advice on reducing the selection pressure for anthelmintic resistance is generally based around avoidance of selection of pre-existing resistant nematodes by not affording them a competitive advantage over susceptible nematodes. Another major influence is the movement of animals infected with drug resistant parasites, leading to gene flow (Kaplan and Vidyashankar, 2012; Redman et al., 2015; Skuce et al., 2010a). Anthelmintic resistance selection and gene flow pressures vary within, and between, seasons and with changing management; hence in the absence of conclusive evidence in support of any single risk factor, the most pragmatic advice given is to instigate mitigation strategies in response to monitoring the resistance status of individual flocks or herds (Ali and Hennessy, 1996).

*In vivo* methods for the diagnosis of benzimidazole resistance, such as the faecal egg count reduction test (FECRT), confirm anthelmintic efficacy by comparing the number of eggs shed in host faeces before and after treatment (Coles et al., 1992, 2006). However, the FECRT for benzimidazole efficacy requires a lag of 10–14 days between each measurement and has poor sensitivity, in particular when less than 25% of the parasite population is resistant (Martin et al., 1989). *In vitro* bioassays, such as the egg hatch test (EHT), expose parasites to titrated concentrations of thiabendazole and measure the number of first stage larvae that hatch. However, the EHT depends on extraction of freshly voided eggs and also has poor sensitivity at low levels of resistance (Taylor et al., 2002).

Various molecular assays and platforms, including conventional PCR (Kwa et al., 1994), combined use of PCR with a restriction enzyme, allele specific PCR (Elard et al., 1996), cloning and Sanger sequencing analysis (Ghisi et al., 2007), real-time PCR (Alvarez-Sanchez et al., 2005), single strand conformation polymorphism (SSCP) genotyping (Skuce et al., 2010b), and pyrosequence genotyping (Samson-Himmelstjerna, 2006), have been used to identify the isotype 1 β-tubulin SNPs in gastrointestinal nematodes. Of these, only real-time PCR and pyrosequencing can provide a practical estimate of allele frequency from pooled samples in a practical way required for large scale field studies, or routine monitoring.

Pyrosequencing is restricted to short fragments of up to 30 bp, while deep amplicon sequencing using Illumina MiSeq can accurately sequence up to about 400 bp reads when used to study nematode species compositions of mixed populations (Avramenko et al., 2015). The method might, therefore, be more sensitive in detecting low frequencies of resistance mutations than conventional methods; while allowing investigation of all three SNPs involved in benzimidazole resistance at once. Of the available next generation sequencing platforms, Illumina MiSeq is least error prone, and best suited to high throughput (Ali et al., 2019; Avramenko et al., 2019). Moreover, Avramenko et al. (2019) undertook a detailed validation study of deep amplicon sequencing to identify the three resistance associated SNPs in the isotype-1 β-tubulin in six different ovine trichostrongylid nematode species.

In the present study, we report the use of deep amplicon sequencing using Illumina MiSeq for the detection of benzimidazole resistance in *T. circumcincta*. A series of experiments was performed to develop and validate the Illumina MiSeq method for measuring resistance allele frequency. First, the number of PCR cycles to amplify DNA before sequencing was varied to identify any sequence representation bias. Second, the results from Illumina MiSeq of *T. circumcincta* laboratory populations were compared to those from pyrosequencing assays. Third, pools were made from resistance allele-genotyped individual third stage larvae (L_3_) gDNA to validate the Illumina MiSeq assay. Finally, the assay was applied to *T. circumcincta* field populations to identify the frequencies of benzimidazole resistance mutations.

## 2 Materials and methods

### 2.1 Parasite materials

Six experimentally passaged and stored *T. circumcincta* laboratory populations were obtained from the Moredun Research Institute, named 1-S (benzimidazole susceptible), 2-R (benzimidazole resistant), 3-R, 4-S, 5-R, 6-R. Pools of larvae (~200 L_3_) were created, by taking 50 μl aliquots from corresponding dilutions in distilled water of each of the six laboratory populations. Three replicate pools of each *T. circumcincta* laboratory population were used with 25, 30, 35, or 40 first round Illumina MiSeq PCR cycles to assess accuracy and determine any sequence representation bias. Three replicate pools of each *T. circumcincta* laboratory population were used for the comparison of the Illumina MiSeq with the pyrosequencing assays.

To quantitatively validate the identification of benzimidazole susceptible or resistant L_3_, 48 individual 1-S L_3_ and 48 individual 5-R L_3_ were first picked into distilled water. Lysates were then prepared for pyrosequence genotyping of the isotype 1 β-tubulin codon 200 locus. 1  μl of lysate from known genotyped individual L_3_ was used to create three replicates each of five admixtures of homozygous susceptible (S: TTC) and homozygous resistant (R: TAC) L_3_ gDNA.

*T. circumcincta* field populations were derived from ewes and lambs on three neighbouring farms in the south-east of Scotland over multiple time points during 2016 and 2017. Farm 1 was a lowland farm with an open sheep flock of about 370 crossbred ewes; Farm 2 was a lowland farm with an open sheep flock of about 680 crossbred ewes; Farm 3 was an extensive hill farm with a closed sheep flock of about 700 hefted Scottish Blackface ewes. There was no movement of animals, or shared grazing between Farm 1 and Farms 2 or 3, but occasional straying between Farms 2 and 3 would have been possible. The faecal samples were collected per rectum, or freshly voided onto the pasture. Following faecal trichostrongyle nematode egg counting using a salt flotation method with a potential sensitivity of one egg per gram (Christie and Jackson, 1982), coprocultures were set up for the recovery of L_3_ (Coles et al., 1992). Pools of about 700 L_3_ were created, by taking 500 μl aliquots from corresponding dilutions in distilled water for each field sample. Ethical approval was acquired through Veterinary Ethics Review Committee (VERC) at the University of Edinburgh, reference number VERC 10 16 and consent was given by the farms’ managers.

### 2.2 Genomic DNA extraction

gDNA was prepared using 1000 μl Direct PCR lysis reagent (Viagen), 50 μl proteinase K solution (Qiagen), and 50 μl 1M dithiothreitol (DTT). To extract gDNA, 25 μl of the mixture was added to each well of a 96-well plate prior to addition of the L_3_. The plate was placed on a thermocycler to incubate at 60 °C for 2 h to lyse the larvae followed by 85 °C for 15 min to inactivate the proteinase K. For pooled L_3_, the samples were first centrifuged for 2 min at 7200×*g*. The supernatant was discarded, and the pellet re-suspended in 50 μl of lysis buffer, which was then placed on a thermocycler with the same conditions described for individual larvae preparation. Genomic DNA was stored at −80 °C for later use.

### 2.3 Illumina MiSeq deep amplicon sequencing of the T. circumcincta isotype 1 β-tubulin locus

Illumina MiSeq was used to sequence a 276 bp fragment of isotype 1 β-tubulin spanning the F200Y, F167Y, and E198L or E198A SNPs. In the first round PCR, a 276 bp fragment of isotype 1 β-tubulin of *T. circumcincta* was amplified with four forward and four reverse adaptor primers (Supplementary Table S1A). The PCR reaction contained 0.5 μl of KAPA HiFi polymerase (KAPA Biosystems), 0.75 μl dNTPs (10 μM), 5 μl 5X KAPA HiFi Fidelity buffer (KAPA Biosystems), 0.75 μl of each for forward and reverse primer mix (10 mM), 13.25 μl nuclease-free water, and 1 μl template DNA. The thermocycling conditions were 95 °C for 2 min, 35 cycles of 98 °C for 20 s, 60 °C for 15 s, 72 °C for 15 s, then a final extension of 72 °C for 2 min and hold at 10 °C. PCR products were purified with AMPure XP Magnetic Beads (1×) (Beckman coulter, Inc.) using a special magnetic stand (DynaMag) in accordance with the protocols described by Beckman coulter, Inc.

The second round PCR was performed by using 16 forward and 24 reverse barcoded primers. (Supplementary Table S1B). Each sample was amplified using a unique combination of barcode primers. The PCR reaction contained 0.5 μl of KAPA HiFi polymerase (KAPA Biosystems), 0.75 μl dNTPs (10 mM), 5 μl 5X KAPA HiFi Fidelity buffer (KAPA Biosystems), 1.25 μl of each primer (10 μM), 13.25 μl nuclease-free water and 2 μl of the first round PCR product as a template. The thermocycling conditions of the PCR were 98 °C for 45 s, followed by 7 cycles of 98 °C for 20 s, 63 °C for 20 s, and 72 °C for 2 min. PCR products were purified with AMPure XP Magnetic Beads (1×) according to the protocols described by Beckman coulter, Inc.

The pooled library was measured with KAPA qPCR library quantification kit (KAPA Biosystems, USA). The prepared library was then run on an Illumina MiSeq sequencer using a 500-cycle pair end reagent kit (MiSeq Reagent Kits v2, MS-103-2003) at a concentration of 15 nM with addition of 10–15% Phix Control v3 (Illumina, FC-11-2003).

### 2.4 Illumina MiSeq data handling

Reference sequence libraries were generated by aligning the *T. circumcincta* isotype 1 β-tubulin sequences downloaded from the NCBI database (Supplementary Table S2) using Geneious v10.2.5 software (Biomatters Ltd, New Zealand). Overall seventy three reference sequences were chosen to represent the spectrum of sequence diversity of *T. circumcincta* isotype 1 β-tubulin locus. Illumina MiSeq separated all sequence data by population during post-run processing by recognised indices and to generate FASTQ files use in subsequent analysis. MiSeq data analysis was performed with a bespoke pipeline (for (more details Supplementary Data S1) using Mothur v1.39.5 software (Schloss et al., 2009) and the Illumina MiSeq SOP (Kozich et al., 2013). Briefly, raw paired-ends reads were run into the make.contigs command to combine the two set of reads for each population. The command was to extract the sequence and quality score data of FASTQ files, create the complement of the reverse and forward reads, and then join the reads into contigs. Sequence data were removed if they were >500 bp or if the reads were too long, or contained ambiguous bases. The sequence data were then aligned with the *T. circumcincta* reference sequence library (Supplementary Table S2) using the align.seqs command and were removed if they did not match with the *T. circumcincta* isotype 1 β-tubulin locus. The summary.seqs command was used to summarise the 277 bp fragments encompassing parts of the *T. circumcincta* isotype 1 β-tubulin. All of the bulk sequence overlap region was further run on the screen.seqs command to generate the *T. circumcincta* isotype 1 β-tubulin sequences FASTQ file. Once all bulk sequences were classified as *T. circumcincta*, a count list of the consensus sequences of each population was created using the unique.seqs command. The count list was further used to create the FASTQ files of the consensus sequences of each population using the split.groups command (for more details Supplementary Data S1).

### 2.5 Phylogenetic analysis of the T. circumcincta isotype 1 β-tubulin locus with other trichostrongylid nematode species of small ruminants

**Supplementary Fig. S1:**
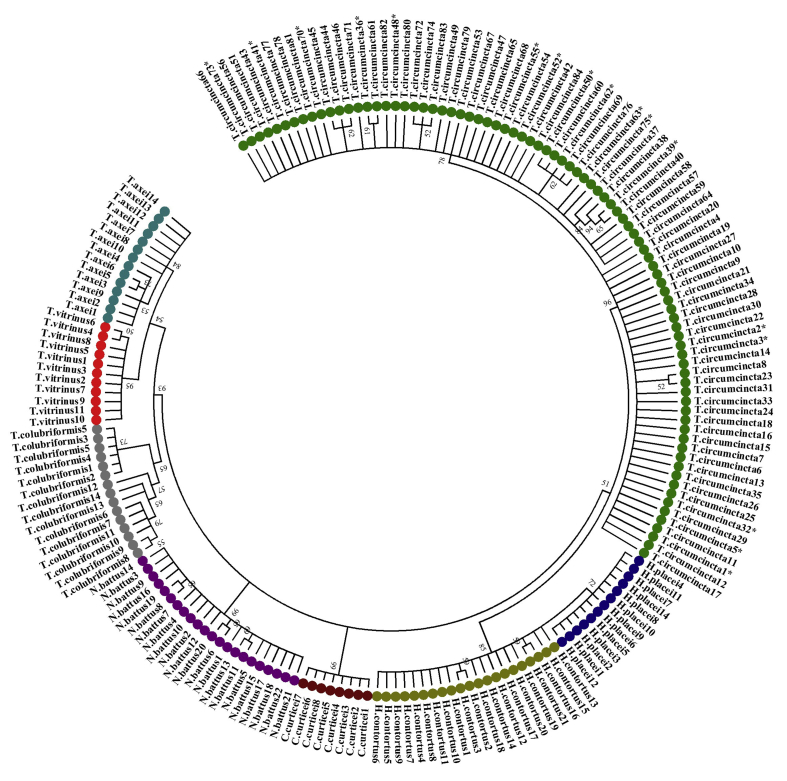
A phylogenetic tree of the consensus sequences of *T. circumcincta* isotype 1 β-tubulin locus and reference sequence library of seven other gastrointestinal nematode species (*Cooperia curticei*, *Trichostrongylus axei*, *Trichostrongylus vitrinus*, *Trichostrongylus colubriformis, Haemonchus placei, Haemonchus contortus* and *Nematodirus battus*) were created. Each species is represented by different colour codes. The tree was constructed by Kimura 2-parameter model of substitution using the Maximum Likelihood method in the MEGA 5.05 software. The program jModeltest 12.2.0 was used to select the appropriate model of nucleotide substitutions for phylogenetic analysis. Branch supports were obtained by 1000 bootstraps of the data. The star shade indicate that those haplotypes are shared between the published and field *T. circumcincta.*

The consensus sequences of *T. circumcincta* isotype 1 β-tubulin were aligned using the MUSCLE alignment tool in Geneious v10.2.5 software (Biomatters Ltd, New Zealand). Details of the sequence data are presented in Supplementary Data S2 and S5. Reference sequence libraries of seven other gastrointestinal nematode species (*Cooperia curticei*, *Trichostrongylus axei*, *Trichostrongylus vitrinus*, *Trichostrongylus colubriformis, Haemonchus placei, Haemonchus contortus* and *Nematodirus battus*) were created from a previously published data set (Ali et al., 2019; Avramenko et al., 2019; Chaudhry et al., 2015; Chaudhry et al., 2015). The obtained isotype 1 β-tubulin sequences of seven species were first aligned in Geneious v10.2.5 (Biomatters Ltd, New Zealand). A phylogenetic tree was constructed by the Kimura 2-parameter model of substitution using the Maximum Likelihood method in the MEGA 5.05 software. The program jModeltest 12.2.0 was used to select the appropriate model of nucleotide substitutions for phylogenetic analysis. Branch supports were obtained by 1000 bootstraps of the data. The sequences of the isotype 1 β-tubulin locus demonstrated separate clustering of *T. circumcincta* in the phylogenetic tree, indicating correct taxonomic assignment of species (Supplementary Fig. S1).

### 2.6 Statistical analysis

The frequency of benzimidazole resistance mutations (F200Y (TAC), F167Y and E198L) was calculated by dividing the number of sequences reads of each population that contained the mutation by the total number of reads. The effect of PCR cycle number on resistance frequency was analysed by running a Kruskal-Wallis rank sum test for each population. Statistical significance in this test would imply a sequence representation bias. A chi-square test was used to determine whether there was a significant difference between allele frequencies of benzimidazole resistant and susceptible individual *T. circumcincta* L_3_ and for the comparison of the Illumina MiSeq deep amplicon sequencing with the pyrosequencing assay. Lin's Concordance Correlation Coefficient was calculated by assessing the agreement level between lamb and ewe samples collected from the three field study farms (DescTools: Tools for Descriptive Statistics).

### 2.7 Pyrosequence genotyping of the T. circumcincta isotype 1 β-tubulin locus

A 276bp fragment of isotype-1 β-tubulin spanning F200Y, F167Y, and E198L or E198A was amplified with the BTUB_FOR and biotin labelled BTUB_REV primers previously described by Silvestre and Humbert (2002) and Skuce et al. (2010a) (Supplementary Table S1A). The PCR reaction contained 0.5 μl of 5 U/μl Taq polymerase (New England Biolabs), 0.5 μl dNTPs (10 mM), 5 μl 10X standard buffer (New England Biolabs), 0.5 μl of each primer (10 μM), 42 μl nuclease-free water and 1 μl DNA template. The thermocycling parameters were 95 °C for 5 min, 35 cycles of 95 °C for 1 min, 60 °C for 1 min, and 72 °C for 1 min, then a final extension at 72 °C for 15 min and hold at 10 °C. Following PCR amplification, the relative frequency of the F200Y, F167Y, and E198L or E198A SNPs were determined by separate pyrosequencing assays using the PyroMark ID system using the allele quantification (AQ) mode of the PSQ 96 single nucleotide position software (Biotage, Sweden). Pyrosequencing primers have been successfully used to genotype the *T. circumcincta* isotype 1 β-tubulin locus from individual larvae using SNP mode (Skuce et al., 2010a). In our hands there are some technical limitation to use this method on pooled samples, therefore, new pyrosequencing primers were designed to target each mutation in six populations (Supplementary Table S1). PyroMark Q96 ID reagents (Qiagen) were used according to the user manual. The base dispensation was set to GTAGCTGTC for codon 200, GTAGCTCA for codon 167, and GATATCGA for codon 198. Peak heights of samples with an unknown number of larvae or individual larvae were measured either in AQ or SNP mode of the PSQ 96 single nucleotide position software previously described by Chaudhry et al. (2015).

## 3 Results

### 3.1 The effect of first round PCR cycle number on the frequency of the isotype 1 β-tubulin locus SNPs in larval pools

**Fig. 1:**
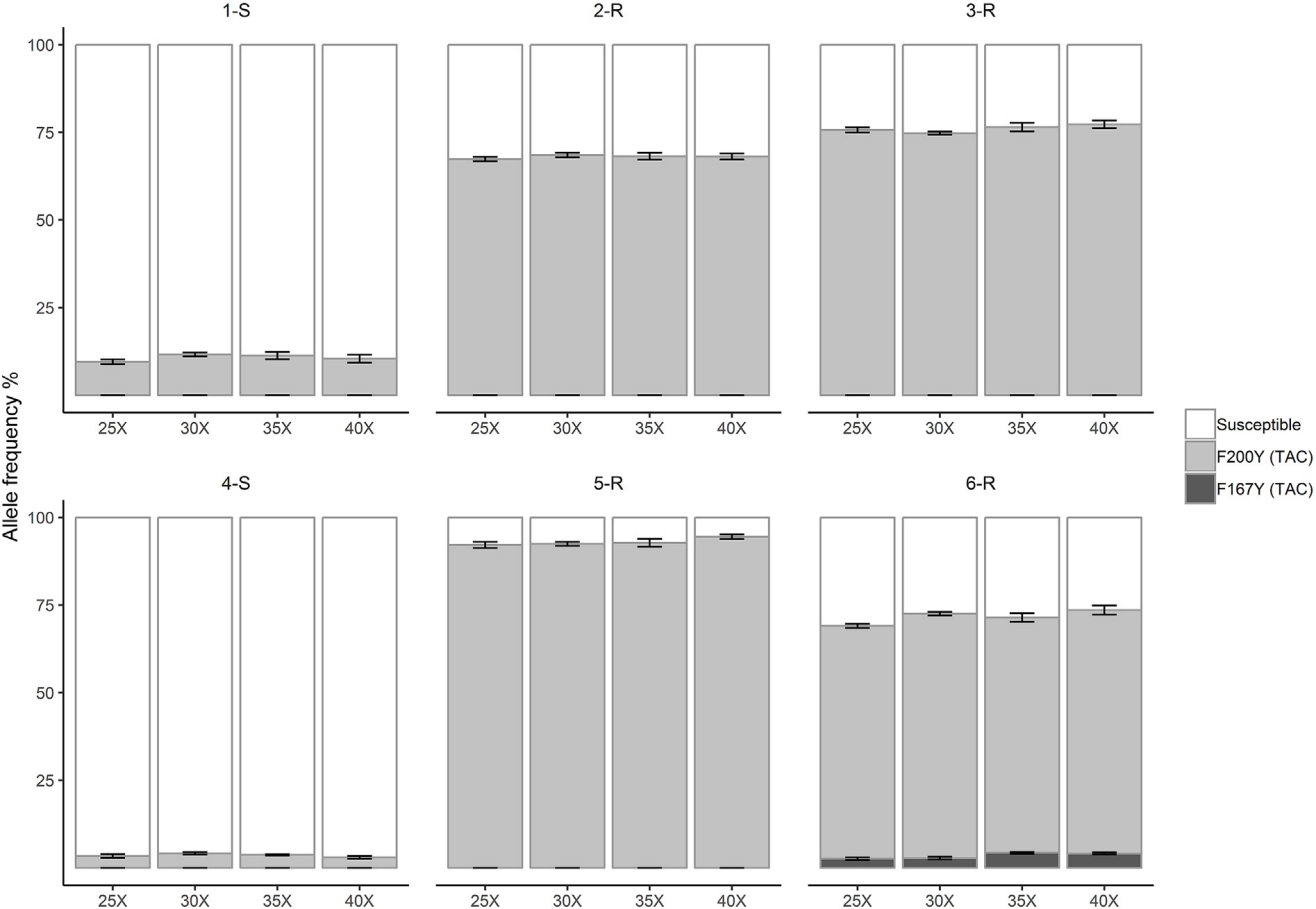
Average frequency of isotype 1 β-tubulin locus SNPs using four different PCR cycles for six *T. circumcincta* laboratory populations. This data was generated from pooled samples with about 200 L_3_. The F200Y (TAC) resistance alleles were identified in all six populations with different frequencies. The F167Y (TAC) was found only in the 6-R population (Supplementary Table S3). Dark grey shade indicates F167Y (TAC), medium grey indicates F200Y (TAC) and white indicates susceptible alleles. X-axis represents the four PCR cycles numbers (25X, 30X, 35X, 40X) and Y-axis represents the allele frequencies. Error bars represent the standard error of the mean.

To assess accuracy and determine any sequence representation bias, three replicates of each mock pool were amplified from the six *T. circumcincta* laboratory populations using four different numbers of first round PCR cycles. According to a Kruskal-Wallis rank sum test (performed separately for each of the mock populations), differences in the proportion of resistant or susceptible alleles at each SNP with the number of PCR cycles were not statistically significant (overall *H*_(3)_<0.2, *p*>0.9). Therefore, no sequence representation bias was observed in the estimation of benzimidazole resistance allele frequencies of the laboratory populations. The F200Y (TAC) SNP was found at different frequencies of between 67.2% and 94.5% in the four phenotypically benzimidazole resistant (2-R, 3-R, 5-R and 6-R) populations and consistently between 3% and 11.6% in the two phenotypically benzimidazole susceptible (1-S and 4-S) populations (Fig. 1 and Supplementary Table S3). The benzimidazole resistance-associated F167Y (TAC) SNP was identified at a frequency of between 2% and 4.3% in the 6-R population (Fig. 1 and Supplementary Table S3). The benzimidazole resistance associated E198L (TTA) and E198A (GCA) SNPs were not detected in any of the populations. Subsequent work used 30 cycles for the first round PCR.

### 3.2 Comparison of the Illumina MiSeq deep amplicon sequencing and pyrosequencing assays to determine the frequency of benzimidazole resistance SNPs

**Fig. 2:**
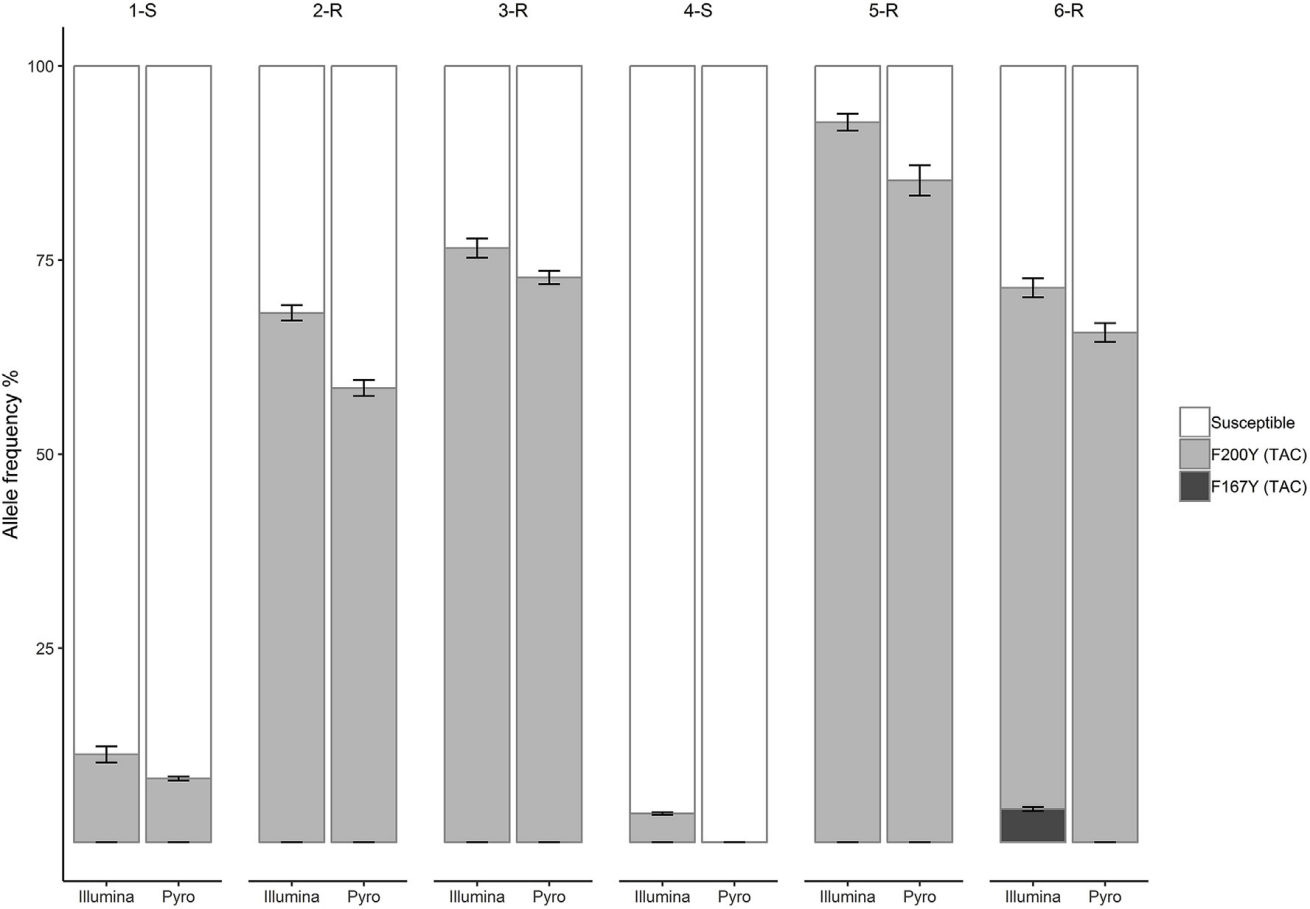
Average frequency of isotype 1 β-tubulin locus SNPs in six *T. circumcincta* laboratory populations, determined by Illumina MiSeq and pyrosequencing. This data was taken from pooled samples with about 200 L_3_. Dark grey shading indicates the F167Y (TAC) SNP, medium grey shading indicates the F200Y (TAC) SNP and white indicates susceptible alleles. Error bars represent the standard error of the mean.

For the comparison of the Illumina MiSeq with the pyrosequencing assay, three replicates each mock pool were taken from six *T. circumcincta* laboratory population. While there were variations between the results of the two methods (with the Illumina MiSeq method apparently more sensitive at low SNP frequencies), differences in the frequency of the benzimidazole resistance SNPs determined by Illumina MiSeq and pyrosequencing were not statistically significant (Chi-square test: χ^2^_(4)_ = 3.962, *p* = 0.4112). The F200Y (TAC) SNP was found at different frequencies in the different populations of between 11.3% and 94.5% in the Illumina MiSeq and 8.2% and 85.3% in the pyrosequencing assay (Fig. 2 and Supplementary Table S4). The F200Y (TAC) and F167Y (TAC) SNPs were only detected at low frequencies of 3.7% and 4.5% by Illumina MiSeq in the 4-S and 6-R populations, respectively, but were not identified by the pyrosequencing assay (Fig. 2 and Supplementary Table S4). The benzimidazole resistance associated E198L (TTA) and E198A (GCA) SNPs were not detected by either method in any of the populations.

### 3.3 Validation of the proportions of benzimidazole resistant and susceptible individual T. circumcincta L_3_

**Fig. 3:**
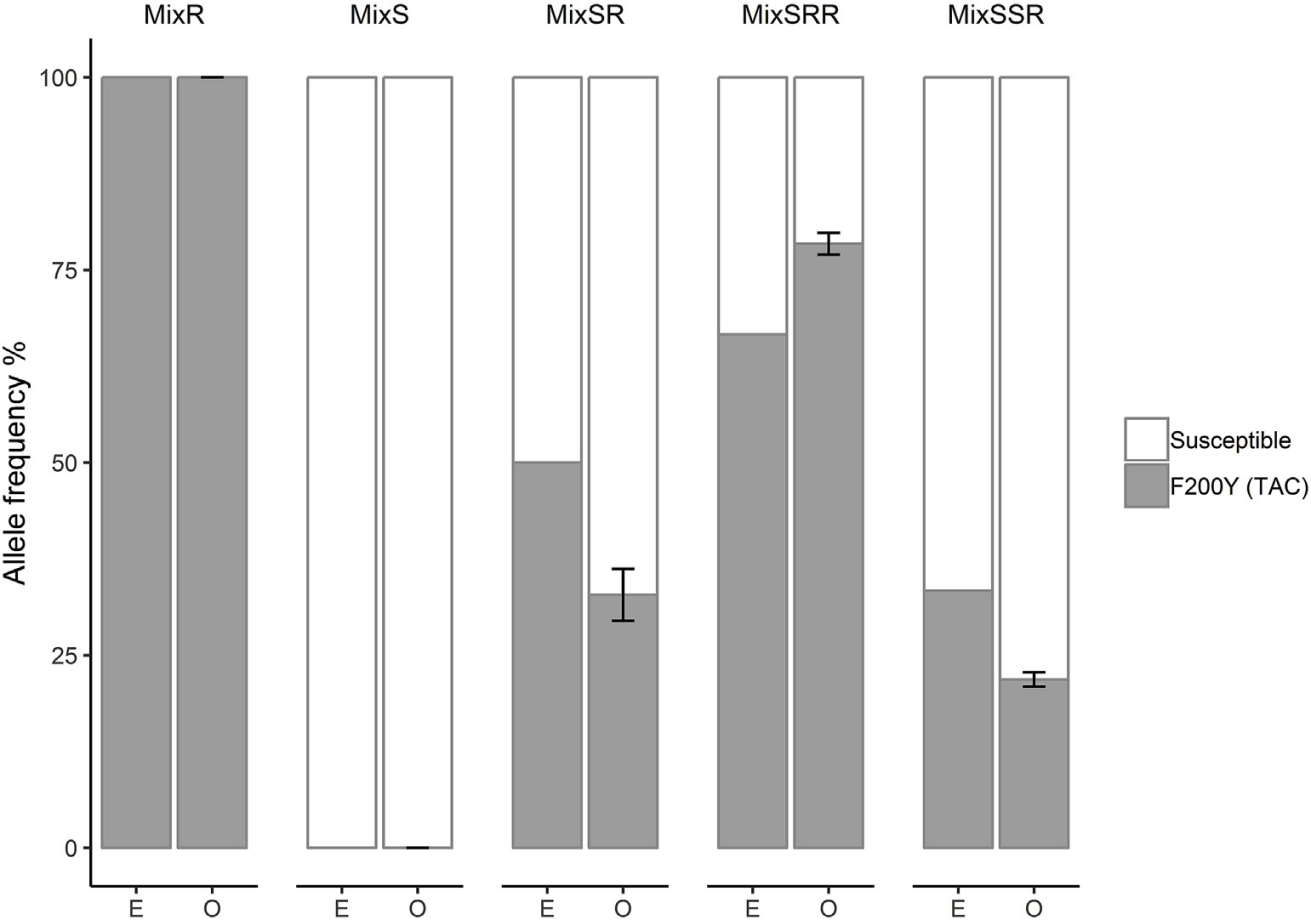
Average frequency of the isotype 1 β-tubulin locus F200Y (TAC) SNP in mock pools was made from different mixing of pyrosequence genotyped 1-S individual homozygous susceptible and 5-R homozygous resistant *T. circumcincta* L_3_. The symbol mix represents the mixing of resistant-R and susceptible-S alleles. In the X-axis, E represents the expected allele frequencies and O represents the observed allele frequencies based on Illumina MiSeq.

To assess the accuracy of the Illumina MiSeq platform in identifying the frequencies of benzimidazole susceptible 1-S and resistant 5-R individual L_3_ were first genotyped by pyrosequencing for the F200Y mutation. This identified homozygous susceptible (S: TTC) and homozygous resistant (R: TAC) alleles. Three replicates each of known admixtures were created of individual L_3_ gDNA from populations that had been pyrosequence genotyped, allowing gDNA pools to be produced with known allele frequencies at the isotype 1 β-tubulin codon 200 locus and used to validate the Illumina MiSeq method. There was no statistically significant difference between the expected and observed frequencies of susceptible and resistance alleles in a chi-square test, which suggests that the Illumina MiSeq method was accurate in measuring resistance allele frequencies (Chi-square test; mixS: χ^2^_(1)_<0.001, *p* = 1; mixR: exact match; mixSR: χ^2^_(1)_<0.001, *p* = 1; mixSRR: χ^2^_(1)_ = 0.057, *p* = 0.845; mixSSR: χ^2^_(1)_ = 0.065, *p* = 0.799) (Fig. 3, Supplementary Table S5). For the pools of 100% susceptible or resistance alleles (MixS(F200Y-TTC)-100, MixR (F200Y-TAC)-100], the expected and observed results were perfectly matched (Fig. 3). For pools of susceptible and resistance alleles [MixSR (F200Y-TTC/TAC)-, MixSRR (F200Y-TTC/TAC)-MixSSR(F200Y-TTC/TAC)-], the expected allele frequencies were 50/50, 33/67, 67/33 and observed results were nearly accurate (68/32, 22/78, 77/23), with not-significant variations between replicates (Fig. 3 and Supplementary Table S5).

### 3.4 Detection of isotype 1 β-tubulin locus SNPs in T. circumcincta field populations using the Illumina MiSeq deep amplicon sequencing method

**Fig. 4:**
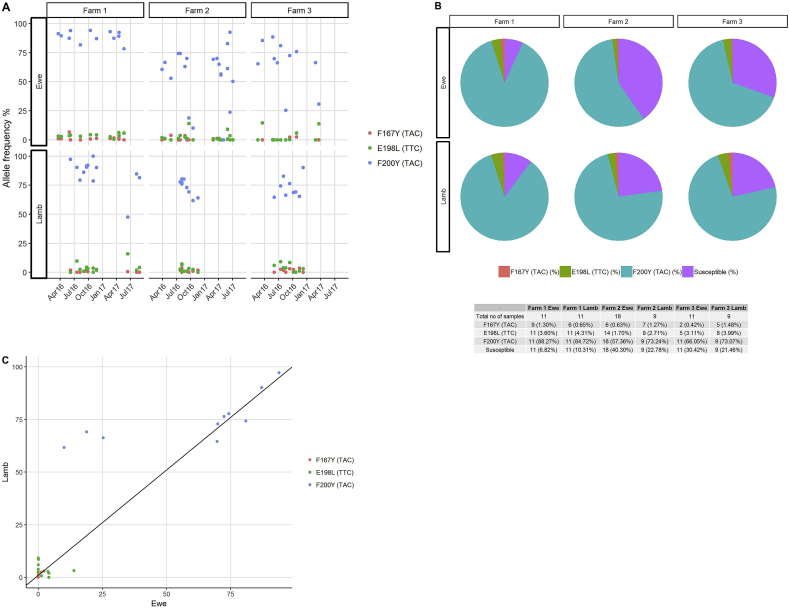
Allele frequencies of isotype 1 β-tubulin locus SNPs in field populations of *T. circumcincta*. Red colour indicates F167Y (TAC), green indicates E198L (TTA) and blue indicates F200Y (TAC) and purple indicates susceptible alleles. Fig. 4A: dots represent the resistance allele frequencies for each sampling time-point. The data shows the three farms with date of sample collection on the X-axis and the Y-axis representing the allele frequencies. Fig. 4B: the pie charts show the overall prevalence of the isotype 1 β-tubulin locus SNPs in ewes and lambs on each farm. The prevalence of SNPs and combinations of SNPs at farm level are shown in the table below the figure. (For interpretation of the references to colour in this figure legend, the reader is referred to the Web version of this article.)

The Illumina MiSeq deep amplicon sequencing was used to detect the frequency of isotype 1 β-tubulin locus SNPs in field samples, In total, field samples from 48 ewes and 37 lambs from three farms were collected at multiple time points in 2016 and 2017. Those samples yielding fewer than 2000 reads (implying insufficient gDNA for accurate amplification) were removed from the analysis, leaving 43 ewe and 31 lamb samples (Supplementary Table S6). The allele frequencies of benzimidazole resistance mutations are shown in Fig. 4. For Farm 1, the F200Y resistance allele frequency was between 78% and 100% in both ewes and lambs. For Farm 2, the F200Y resistance allele frequency was between 61.7% and 80.2% in the lambs, but varied between 10% and 92% in the ewes. For Farm 3, the F200Y resistance allele frequency was between 64.6% and 90.2% in the lambs, but varied between 25.3% and 88.5% in the ewes. The F167Y allele frequency was between 0.5% and 6.6% on all three farms. The E198L (TTA) allele frequency was between 0.1% and 13.9% on all three farms (Fig. 4A). In terms of overall prevalence, the F200Y (TAC) resistance-associated SNP was found on all three farms in each of the ewe and lamb samples collected at different time-points. The F167Y (TAC) resistance associated SNP was detected in 9/12 ewe and 7/13 lamb time-point samples on Farm 1; in 7/20 ewe and 7/9 lamb time-point samples on Farm 2; and in 2/11 ewe and 5/9 lamb time-point samples on Farm 3. The E198L (TTA) resistance associated SNP was detected in 12/12 ewe and 11/13 lamb time-point samples on Farm 1; 15/20 ewe and 9/9 lamb time-point samples on Farm 2; and 5/11 ewe and 8/9 lamb time-point samples on Farm 3 (Fig. 4B). Overall, the statistical correlation [(r_c_ = 0.983 (0.791–0.946)] shows a high level of agreement between lamb and ewe samples from each of the three farms (Fig. 4C).

## 4 Discussion

Genotypic markers for anthelmintic resistance and sensitive and accurate platforms with which to measure them are needed for accurate surveillance to assess, and mitigate, the impacts of climate change, animal husbandry practices and pasture management on the emergence and spread of anthelmintic resistance mutations. *In vivo* bioassays such as the egg hatch and larval development (Coles et al., 1992) tests for benzimidazole resistance may be influenced by extrinsic or separate genetic factors governing traits such as fitness in the test environment, or by variation in the test conditions. Furthermore, the mechanisms of drug resistance in eggs and developing larvae may differ from those in parasitic stages (Kotze et al., 2012). The *in vivo* faecal egg count reduction test is influenced by independent host factors, such as those affecting drug bioavailability (Ali and Hennessy, 1996), or by the accuracy or drug administration. Phenotypic assays are also influenced by the number of genes involved and by the dominance or recessiveness of the trait; hence are poor in the estimation of the frequency of resistance alleles, in particular when the frequency is low. Hence, they may not accurately reflect the emergence and spread of anthelmintic resistance mutations in field populations of gastrointestinal nematodes at early stages of development.

Validated genotypic markers for anthelmintic resistance are only currently available for the benzimidazoles (Geary et al., 1992b), albeit marker discovery is a research priority for the imidazothiazole, macrocyclic lactone and amino-acetonitrile derivative broad spectrum anthelmintic drug groups (Doyle et al., 2019). In this study, we used the isotype 1 β-tubulin SNPs to develop a proof of concept method to examine the frequency of alleles in gastrointestinal nematode populations, with reference to better understanding of the impact of environmental factors on the emergence and spread of anthelmintic resistance. Deep amplicon sequencing by Illumina MiSeq offers potential for greater practicality that is required for field investigations than pyrosequencing, because: fewer replicates are needed to overcome the chance of quality control failure; and separate pyrosequencing runs are required for analysis of each isotype 1 β-tubulin SNP, spanning codons 167, 198 and 200, whereas the Illumina MiSeq platform allows for the determination of multi-allelic polymorphisms spanning an approximately 400 bp locus. Illumina MiSeq involves two short PCRs and two rounds of product purification before sequencing, and in our hands provided informative read depths in at least 384 samples (four 96-well plates) in a single library; hence is most practical for use in high throughput scenarios (Avramenko et al., 2019). In this assay, amplicons were generated from *T. circumcincta* laboratory and field L_3_ populations and sequenced in depth using the Illumina MiSeq platform. The resultant sequences were then bioinformatically assigned to the corresponding parasite species on the basis of sequence identity when compared against *T. circumcincta* isotype 1 β-tubulin consensus sequence taxonomy library. The allele frequencies of the three isotype 1 β-tubulin F200Y (TAC), F167Y and E198L SNPs for the *T. circumcincta* populations were analysised using a bioinformatic pipeline described in material and methods section 2.4. Briefly, raw paired-ends reads were run into the make.contigs command, to remove the ambigious bases. The sequence data were aligned with the *T. circumcincta* isotype 1 β-tubulin consensus sequence library using the align.seqs command and the un-aligned sequences were discarded. All the bulk sequence of 276 bp fragments overlaps the same region were run on the screen.seqs command to generate the isotype 1 β-tubulin matched sequences of *T. circumcincta*. Finally, the analysis of the three isotype 1 β-tubulin F200Y (TAC), F167Y and E198L SNPs for the *T. circumcincta* populations was performed using Geneious v10.2.5 software. Based on these commands, we are confident that only *T. circumcincta* DNA was amplified. Furthermore the field samples showed the predominance of *T. circumcincta* in faecal samples from grazing ewes and lambs in each farm (data on file). The other gastrointestinal nematode species identified on the three farms were *Oesophagostomum venulosum*, *Cooperia curticei*, *Trichostrongylus axei*, *Trichostrongylus vitrinus* and *Trichostrongylus unclassified* (data on file).

We validated the Illumina MiSeq method to examine benzimidazole resistance allele frequencies in mock population pools of *T. circumcincta* by: assessing sequence representation bias in the isotype 1 β-tubulin locus; comparing the results of Illumina MiSeq and pyrosequencing; and applying the method to populations containing known proportions of resistant and susceptible L_3_. We identified no significant variation in the frequency of resistance alleles with the number of first round PCR cycles in any of the six reference *T. circumcincta* populations; hence showed no sequence representation bias (Avramenko et al., 2015) arising from the number of first round PCR cycles, or from potential variation in the efficiency of other library preparation steps, or from any inaccuracy in the estimation of the number of about 200 L_3_ making up each mock population pool. The Illumina MiSeq method showed a higher F200Y (TAC) resistance allele frequency than pyrosequencing in each of the six reference *T. circumcincta* populations, but the differences were not shown to be statistically significant. This might imply that the Illumina MiSeq method is more sensitive than pyrosequencing, but true determination of sensitivity would require the genotyping the individual L_3_ making up each of the mock population pools as described by (Avramenko et al., 2019). Five mock population pools containing different estimated proportions of homozygous F200Y (TAC) resistance mutations were generated by mixing fixed amounts of pyrosequence genotyped individual L_3_ gDNA, derived from susceptible and drug selected resistant laboratory *T. circumcincta* populations. For the pools that were 100% resistant or 100% susceptible, the observed allele frequencies based on Illumina MiSeq were in perfect agreement with expected allele frequencies. For pools with 67%, 50%, and 33% resistance alleles, differences between the expected and observed frequencies of the F200Y (TAC) mutation were not statistically significant (p > 0.9), with little variation between replicates, providing support for the accuracy of the Illumina MiSeq method in determining the frequency of resistance alleles in a gastrointestinal nematode parasite population. The small amount of variation between observed and expected allele frequencies could have arisen in the creation of the mock pools using only 1 μl of low concentration gDNA derived from about 90 individual L_3_.

Gastrointestinal nematodes impact heavily on animal welfare and production, hence there is a need to understand the population genetics of anthelmintic resistance (Gilleard and Beech, 2007). Having validated the Illumina MiSeq platform using mock pools of laboratory *T. circumcincta* isolates, we applied the method to field samples collected from ewes and lambs on three farms, each highlighting different aspects of sheep management and approaches to parasite control. This was undertaken as proof of concept to explore the possibilities for the application of a high throughput practical method to determine the proportions of resistance alleles in particular gastrointestinal nematode parasite species within mixed species field populations.

We have further examined the frequency of the total of the F200Y (TAC), E198L (TTC) and F167Y (TAC) mutations with reference to resistance allele frequencies in the field *T. circumcincta* populations. A different pattern of resistance allele frequency over time emerged on Farm 1 compared with Farm 2 and Farm 3. On farm 1, the overall frequencies of resistance alleles in ewes and lambs were about 94% and 90%, respectively, whereas the overall frequencies of resistance alleles in ewes and lambs on Farm 2/Farm 3 were 58%/70% and 77%/79%, respectively. Together these results indicate high frequencies of benzimidazole resistance alleles on all three farms, having nearly reached genetic fixation on Farm 1. This scenario highlights an opportunity to use the results of high throughput anthelmintic resistance allele genotyping to investigate the genetic selection pressures and question management practices that might have impacted on them. For example, there had been little uptake of mitigation strategies on Farm 1 following the first diagnosis of anthelmintic resistance in 2000 (data on file); whereas Farm 2 had ceased anthelmintic treatments of periparturient ewes and implemented alternative grazing management with cattle following the diagnosis of anthelmintic resistance in 2001 (Sargison et al., 2001); and Farm 3 had cautiously reduced the anthelmintic treatment frequency of ewes and lambs and adopted refugia management strategies involving delayed treatments of lambs moved onto safe in-bye pastures, and avoidance of whole group treatments following the diagnosis of anthelmintic resistance in 2005 (Sargison et al., 2005). It is intriguing to consider the genetic fixation of benzimidazole resistance alleles on Farm 1 in the context of the sheep flock being open, having introduced replacement ewes annually that presumably (based on our observations in farm 2 and 3) harboured benzimidazole susceptible genotypes. The gene flow of alleles conferring susceptibility to benzimidazole drugs on Farm 1 may have been halted by strategic treatments of the introduced animals with amino-acetonitrile derivative, or anthelmintic drug combinations. This failure to introduce susceptible genotypes might challenge the application of dogma surrounding quarantine treatments of introduced animals with anthelmintic drug combinations (Leathwick and Besier, 2014). However, quarantine treatments are needed to mitigate the impact of resistance to each of the broad spectrum anthelmintic drug classes at once, hence the strategy of allowing the introduction of susceptible genotypes would only be appropriate if the population genetics of resistance alleles for each group were to be understood at an individual farm level (Greer et al., 2009). This would require molecular markers for resistance to each of the anthelmintic drug groups. Once genetic fixation of resistance alleles has occurred, the probabilities of success of key mitigation strategies, such as the use of anthelmintic drug combinations (Leathwick et al., 2015), or refugia management involving targeted selective treatments (Greer et al., 2009), in achieving reversion to drug susceptibility are reduced.

On Farm 2 and Farm 3, where genetic fixation had not been reached, there were some between sample variations in the frequency of benzimidazole resistance alleles. Some of this would have been due to stochastic effects caused by different numbers of *T. circumcincta* L_3_ in the sample pools, but the observation nevertheless highlights opportunities to map sustained trends in the frequency of resistance alleles, indicating genetic drift or bottlenecks, to climatic variation and specific practices, such as anthelmintic drug treatments and grazing management. This was unrewarding in this case, where no benzimidazole treatments were administered in Farm 2 and Farm 3, and possibly because the frequency of resistance alleles was already too high to detect significant changes; showing a need for molecular surveillance starting before resistance reaches a level where it can be phenotypically identified. The similarities that were observed in the resistance SNP frequencies generated from parasite populations harvested from individual co-grazed animals suggest that it is possible to screen parasite communities on pasture for resistance SNPs from a small number of hosts.

In summary, there is a pressing need for high throughput methods to determine the frequency of anthelmintic resistance conferring mutations, or markers, in field populations of gastrointestinal nematodes. We have demonstrated the feasibility and practicality of deep amplicon sequencing by Illumina MiSeq in this regard, using benzimidazole resistance in *T. circumcincta* as proof of concept, and have considered how knowledge of resistance allele frequencies in field populations might be used to build hypotheses on selection pressures and inform mitigation strategies. The method has previously been applied to other gastrointestinal nematode species, and multiplexed to study benzimidazole resistance co-infections (Avramenko et al., 2019). Once markers become available, the method might also be used to study single or multiple mutations conferring resistance to other anthelmintic drug groups, and combined with phylogenetic tools to show their emergence and spread.

## Conflicts of interest

The authors declare that they have no competing interests.
