# Supplementary material for: Development of amplicon sequencing for the analysis of benzimidazole resistance allele frequencies in field populations of gastrointestinal nematodes
Source: Int J Parasitol Drugs Drug Resist. 2019 Aug 13;10:92–100. doi: 10.1016/j.ijpddr.2019.08.003 (PMC6708983; doi:10.1016/j.ijpddr.2019.08.003)
Supplement: Multimedia component 1 [file mmc1.docx]

**Supplementary Table S1A**: Primer sequences used for this study. Presented results of pyrosequencing used the P167 TC, P198 TC, and P200 TC primers. Illumina adapters are the Nextera transposase adapters, and for each direction, there are four versions with a different number of random nucleotides (N).

| Primer | Purpose | Sequence, 5’-3’ | Reference |
| --- | --- | --- | --- |
| BTUB_FOR | Amplifying β-tubulin for pyrosequencing | CCAAAATTCGCGAGGAGTA | Skuce *et al*. 2010 |
| BTUB_REV (biotin) | Amplifying β-tubulin for pyrosequencing | 5Bioag/TTTCAAGGTGCGGAAGCAGA |  |
| P167 TC | Pyrosequencing | ATAGAATCATGGCTTCAT | Unpublished |
| P198 TC | Pyrosequencing | GGTWGAAAAYACCGAYK |  |
| P200 TC | Pyrosequencing | GAAAAYACCGATGAAACRT |  |
| BTUB_FOR with Illumina adapter | Amplifying β-tubulin for Illumina Mi-seq | TCGTCGGCAGCGTCAGATGTGTATAAGAGACAGCCAAAATTCGCGAGGAG*T*A | Oligonucleotide sequences © 2018 Illumina, Inc. All rights reserved |
| BTUB_FOR with Illumina adapter (1N) | Amplifying β-tubulin for Illumina Mi-seq | TCGTCGGCAGCGTCAGATGTGTATAAGAGACAGNCCAAAATTCGCGAGGAG*T*A |  |
| BTUB_FOR with Illumina adapter (2N) | Amplifying β-tubulin for Illumina Mi-seq | TCGTCGGCAGCGTCAGATGTGTATAAGAGACAGNNCCAAAATTCGCGAGGAG*T*A |  |
| BTUB_FOR with Illumina adapter (3N) | Amplifying β-tubulin for Illumina Mi-seq | TCGTCGGCAGCGTCAGATGTGTATAAGAGACAGNNNCCAAAATTCGCGAGGAG*T*A |  |
| BTUB_REV with Illumina adapter | Amplifying β-tubulin for Illumina Mi-seq | GTCTCGTGGGCTCGGAGATGTGTATAAGAGACAGTTTCAAGGTGCGGAAGCA*G*A |  |
| BTUB_REV with Illumina adapter (1N) | Amplifying β-tubulin for Illumina Mi-seq | GTCTCGTGGGCTCGGAGATGTGTATAAGAGACAGNTTTCAAGGTGCGGAAGCA*G*A |  |
| BTUB_REV with Illumina adapter (2N) | Amplifying β-tubulin for Illumina Mi-seq | GTCTCGTGGGCTCGGAGATGTGTATAAGAGACAGNNTTTCAAGGTGCGGAAGCA*G*A |  |
| BTUB_REV with Illumina adapter (3N) | Amplifying β-tubulin for Illumina Mi-seq | GTCTCGTGGGCTCGGAGATGTGTATAAGAGACAGNNNTTTCAAGGTGCGGAAGCA*G*A |  |
