# Supplementary material for: Development of amplicon sequencing for the analysis of benzimidazole resistance allele frequencies in field populations of gastrointestinal nematodes
Source: Int J Parasitol Drugs Drug Resist. 2019 Aug 13;10:92–100. doi: 10.1016/j.ijpddr.2019.08.003 (PMC6708983; doi:10.1016/j.ijpddr.2019.08.003)
Supplement: Multimedia component 3 [file mmc3.docx]

**Supplementary Table 2**: Reference sequences library of *T. circumcincta* isotype 1 β-tubulin generated from the NCBI database.

| **Isotype 1 β-tubulin** | **GeneBank accession number** |
| --- | --- |
| *T. circumcincta* | FN599038.1, FN599039.1, KF483638.1, KP204106.1, KF483641.1, KF483648.1, KF483626.1, KF483619.1, GQ910869.1, FN599034.1, GQ910873.1, FN599036.1, KF483625.1, KF483657.1, KF483640.1, KF483656.1, KF483655.1, FN599037.1, KF483618.1, KF483620.1, KF483637.1, KF483654.1, KF483621.1, KF483653.1, KF483647.1, GQ910859.1, GQ869653.1, GQ910858.1, GQ910853.1, KF483651.1, KF483652.1, KF483636.1, KF483616.1, GQ910868.1, FN599052.1, FN599043.1, KP204108.1, KF483635.1, KF483642.1, KF483627.1, KF483624.1, KF483650.1, KF483634.1, KF483645.1, KF483649.1, KP204104.1, KF483643.1, FN599046.1, KF483628.1, KF483622.1, KF483629.1, FN599047.1, KF483644.1, KF483633.1, FN599041.1, KF483631.1, KF483615.1, KF483630.1, KF483623.1, KF483632.1, KF483617.1, GQ910863.1, GQ910864.1, GQ910860.1, KP204103.1, KP204099.1, KP204102.1, KP204105.1, KF483639.1, KP204100.1, KP204101.1, KF483646.1, FN599053.1, KP204098.1, KP204107.1, FN599049.1 |
