# Supplementary material for: Development of amplicon sequencing for the analysis of benzimidazole resistance allele frequencies in field populations of gastrointestinal nematodes
Source: Int J Parasitol Drugs Drug Resist. 2019 Aug 13;10:92–100. doi: 10.1016/j.ijpddr.2019.08.003 (PMC6708983; doi:10.1016/j.ijpddr.2019.08.003)
Supplement: Multimedia component 5 [file mmc5.docx]

**Supplementary Table S4:** Benzimidazole resistance allele frequencies in pools of *T. circumcincta* laboratory populations have been determined by two methods: Illumina Mi-seq and pyrosequencing genotyping. Both F167Y (TAC) and F200Y (TAC) mutations were identified by Illumina Mi-seq and only F200Y (TAC) mutation was found by pyrosequencing.

| **Method** | **Sample**  **ID** | **Illumina MiSeq reads**  **(Pre filtered)** | **Illumina MiSeq reads**  **(Post filtered)** | **Mean no. of susceptible reads (Post filtered)** | **Mean no. of resistant reads (Post filtered)** | **F167Y (%)** | | **F200Y (%)** | |
| --- | --- | --- | --- | --- | --- | --- | --- | --- | --- |
|  |  |  |  |  |  | TTC | TAC | TTC | TAC |
| **Illumina MiSeq** | 1-S | 8708 | 7842 | 6902 | 940 | 100.00 | 0.00 | 88.69 | 11.31 |
|  | 2-R | 10947 | 10397 | 3270 | 7127 | 100.00 | 0.00 | 31.82 | 68.18 |
|  | 3-R | 12580 | 12556 | 2938 | 9618 | 100.00 | 0.00 | 23.46 | 76.54 |
|  | 4-S | 5650 | 4124 | 3980 | 144 | 100.00 | 0.00 | 96.51 | 3.70 |
|  | 5-R | 5661 | 5602 | 393 | 5209 | 100.00 | 0.00 | 7.23 | 92.77 |
|  | 6-R | 11678 | 11113 | 3577 | 7536 | 95.72 | 4.28 | 32.85 | 67.15 |
| **Pyrosequencing** | 1-S |  | Not applicable | | | 100.0 | 0 | 91.8 | 8.2 |
|  | 2-R |  |  |  |  | 100.0 | 0 | 41.5 | 58.5 |
|  | 3-R |  |  |  |  | 100.0 | 0 | 27.2 | 72.8 |
|  | 4-S |  |  |  |  | 100.0 | 0 | 100.0 | 0.0 |
|  | 5-R |  |  |  |  | 100.0 | 0 | 14.7 | 85.3 |
|  | 6-R |  |  |  |  | 100.0 | 0 | 34.3 | 65.7 |
