# Supplementary material for: Development of amplicon sequencing for the analysis of benzimidazole resistance allele frequencies in field populations of gastrointestinal nematodes
Source: Int J Parasitol Drugs Drug Resist. 2019 Aug 13;10:92–100. doi: 10.1016/j.ijpddr.2019.08.003 (PMC6708983; doi:10.1016/j.ijpddr.2019.08.003)
Supplement: Multimedia component 6 [file mmc6.docx]

**Supplementary Table S5:** Mean frequency of the F200Y (TAC) mutation for benzimidazole resistance, made using pyrosequence genotyped individual larvae from phenotypically benzimidazole susceptible (S-1) and resistant (5-R) *T. circumcincta* laboratory populations*.* The expected frequency is calculated based on how the pools were made.

| **Pooled samples** | **Illumina MiSeq reads**  **(Pre filtered)** | **Illumina MiSeq reads**  **(Post filtered)** | **Mean no. of susceptible reads (Post filtered)** | **Mean no. of resistant reads (Post filtered)** | **Observed**  **frequency**  **F200Y (%)** | | | | **Expected**  **frequency**  **F200Y (%)** | |
| --- | --- | --- | --- | --- | --- | --- | --- | --- | --- | --- |
|  |  |  |  |  | | TTC | TAC | TTC | | TAC |
| MixS* | 7739 | 7195 | 7195 | 0 | | 100.0 | 0.0 | 100 | | 0 |
| MixR^¥^ | 8436 | 8147 | 0 | 8147 | | 0.0 | 100.0 | 0 | | 100 |
| MixSR*^¥^ | 9234 | 8452 | 5731 | 2721 | | 68 | 32 | 50 | | 50 |
| MixSRR*^¥¥^ | 9671 | 9302 | 2006 | 7296 | | 22 | 78 | 33 | | 67 |
| MixSSR**^¥^ | 9056 | 7943 | 6103 | 1840 | | 77 | 23 | 67 | | 33 |

*S: single individual susceptible larvae, ^¥^R: single individual resistant larvae
