# Supplementary material for: Development of amplicon sequencing for the analysis of benzimidazole resistance allele frequencies in field populations of gastrointestinal nematodes
Source: Int J Parasitol Drugs Drug Resist. 2019 Aug 13;10:92–100. doi: 10.1016/j.ijpddr.2019.08.003 (PMC6708983; doi:10.1016/j.ijpddr.2019.08.003)
Supplement: Multimedia component 7 [file mmc7.docx]

**Supplementary Table S6:** Benzimidazole resistance allele frequency of field samples taken from three farms in south-east Scotland from ewes and lambs, monthly over the period of 2016-2017. The F167Y (TAC), E198L (TTA), and F200Y (TAC) mutations are shown.

| **Farm** | **Sheep** | **Date** | **Illumina MiSeq reads**  **(Pre filtered)** | **Illumina MiSeq reads**  **(Post filtered)** | | **Mean no. of susceptible reads (Post filtered)** | | **Mean no. of resistant reads (Post filtered)** | | **F167Y (%)** | | | **E198L (%)** | | | **F200Y (%)** | | |  |
| --- | --- | --- | --- | --- | --- | --- | --- | --- | --- | --- | --- | --- | --- | --- | --- | --- | --- | --- | --- |
|  |  |  |  |  |  | |  | |  | | TTC | TAC | | GAA | TTA | | TTC | TAC | |
| **Farm 1** | ewe | 20/3/16 | 75253 |  | 74093 | | 3504 | | 70589 | | 99.1 | 0.9 | | 96.8 | 3.2 | | 8.8 | 91.2 | |
|  |  | 7/4/16 | 159067 |  | 155344 | | 10619 | | 144725 | | 99.2 | 0.8 | | 97.2 | 2.8 | | 10.5 | 89.5 | |
|  |  | 30/5/16 | 14393 |  | 12562 | | 324 | | 12238 | | 93.4 | 6.6 | | 96.6 | 3.4 | | 12.6 | 87.4 | |
|  |  | 10/8/16 | 9408 |  | 8884 | | 1364 | | 7520 | | 100 | 0 | | 97.0 | 3.0 | | 18.3 | 81.7 | |
|  |  | 13/10/16 | 24947 |  | 23804 | | 196 | | 23608 | | 99.3 | 0.7 | | 95.7 | 4.3 | | 5.8 | 94.2 | |
|  |  | 23/11/16 | 125344 |  | 121067 | | 9123 | | 111944 | | 98.8 | 1.2 | | 95.9 | 4.1 | | 12.9 | 87.1 | |
|  |  | 20/2/17 | 9779 |  | 9078 | | 303 | | 8775 | | 99.0 | 1.0 | | 97.3 | 2.7 | | 7.0 | 93.0 | |
|  |  | 16/3/17 | 45100 |  | 44947 | | 4744 | | 40203 | | 98.4 | 1.6 | | 99.4 | 0.6 | | 12.7 | 87.3 | |
|  |  | 17/4/17 | 44865 |  | 44393 | | 1607 | | 42786 | | 98.9 | 1.1 | | 93.8 | 6.2 | | 10.9 | 89.1 | |
|  |  | 18/4/17 | 82084 |  | 80316 | | 2983 | | 77333 | | 99.5 | 0.5 | | 96.5 | 3.5 | | 7.7 | 92.3 | |
|  |  | 21/5/17 | 42078 |  | 41100 | | 6542 | | 34558 | | 100 | 0 | | 94.2 | 5.8 | | 21.7 | 78.3 | |
|  | lamb | 7/6/16b | 129910 |  | 129105 | | 1165 | | 127940 | | 98.2 | 1.8 | | 100 | 0 | | 2.7 | 97.3 | |
|  |  | 18/7/16 | 13816 |  | 13202 | | 0 | | 13202 | | 100 | 0 | | 90.3 | 9.7 | | 9.7 | 90.3 | |
|  |  | 8/8/16 | 74907 |  | 74071 | | 13361 | | 60710 | | 100 | 0 | | 97.4 | 2.6 | | 20.6 | 79.4 | |
|  |  | 20/9/16 | 13233 |  | 13075 | | 455 | | 12620 | | 98.4 | 1.6 | | 95.6 | 4.4 | | 9.5 | 90.5 | |
|  |  | 27/9/16 | 138983 |  | 138303 | | 5505 | | 132798 | | 99.4 | 0.6 | | 96.7 | 3.3 | | 7.9 | 92.1 | |
|  |  | 1/11/16 | 169619 |  | 167890 | | 29691 | | 138199 | | 100 | 0 | | 96.4 | 3.6 | | 21.3 | 78.7 | |
|  |  | 1/11/16b | 8993 |  | 8902 | | 1 | | 8901 | | 100 | 0 | | 100 | 0 | | 0.0 | 100.0 | |
|  |  | 23/11/16b | 229342 |  | 228183 | | 12483 | | 215700 | | 97.5 | 2.5 | | 98.1 | 1.9 | | 9.9 | 90.1 | |
|  |  | 14/6/17 | 32674 |  | 32402 | | 11685 | | 20717 | | 99.4 | 0.6 | | 84.1 | 15.9 | | 52.5 | 47.5 | |
|  |  | 10/8/17 | 2548 |  | 2548 | | 345 | | 2203 | | 100 | 0 | | 98.2 | 1.8 | | 15.3 | 84.7 | |
|  |  | 30/8/17 | 30175 |  | 29910 | | 4268 | | 25642 | | 100 | 0 | | 95.8 | 4.3 | | 18.5 | 81.5 | |
|  |  |  |  |  |  | |  | |  | |  |  | |  |  | |  |  | |
|  |  |  |  |  |  | |  | |  | |  |  | |  |  | |  |  | |
|  |  |  |  |  |  | |  | |  | |  |  | |  |  | |  |  | |
|  |  |  |  |  |  | |  | |  | |  |  | |  |  | |  |  | |
|  |  |  |  |  |  | |  | |  | |  |  | |  |  | |  |  | |
|  |  |  |  |  |  | |  | |  | |  |  | |  |  | |  |  | |
| **Farm 2** | ewe | 29/3/16 | 2133 |  | 2133 | | 802 | | 1331 | | 100 | 0 | | 98.1 | 1.9 | | 39.5 | 60.5 | |
|  |  | 18/4/16 | 13730 |  | 11905 | | 3734 | | 8171 | | 98.8 | 1.2 | | 99.1 | 0.9 | | 33.4 | 66.6 | |
|  |  | 27/5/16 | 67649 |  | 67572 | | 29276 | | 38296 | | 96.2 | 3.8 | | 100 | 0 | | 47.2 | 52.8 | |
|  |  | 14/7/16d | 34526 |  | 34037 | | 8373 | | 25664 | | 100 | 0 | | 98.8 | 1.2 | | 25.8 | 74.2 | |
|  |  | 26/7/16 | 1645 |  | 1608 | | 354 | | 1254 | | 100 | 0 | | 96.2 | 3.8 | | 25.7 | 74.3 | |
|  |  | 26/8/16 | 14045 |  | 13921 | | 4375 | | 9546 | | 96.6 | 3.4 | | 97.8 | 2.2 | | 37.1 | 62.9 | |
|  |  | 7/9/16 | 34069 |  | 32373 | | 9316 | | 23057 | | 100 | 0 | | 98.8 | 1.2 | | 30 | 70 | |
|  |  | 20/9/16d | 12789 |  | 12789 | | 8602 | | 4187 | | 100 | 0 | | 86.1 | 13.9 | | 81.2 | 18.8 | |
|  |  | 17/10/16 | 43962 |  | 43935 | | 39501 | | 4434 | | 100 | 0 | | 100 | 0 | | 89.9 | 10.1 | |
|  |  | 26/2/17b | 29940 |  | 29809 | | 8932 | | 20877 | | 100 | 0 | | 99.2 | 0.8 | | 30.8 | 69.2 | |
|  |  | 22/3/17 | 26732 |  | 25695 | | 7201 | | 18494 | | 98.8 | 1.2 | | 99.3 | 0.7 | | 30.0 | 70.0 | |
|  |  | 5/4/17 | 24950 |  | 24862 | | 8409 | | 16453 | | 98.9 | 1.1 | | 99.9 | 0.1 | | 35.0 | 65.0 | |
|  |  | 17/4/17b | 64708 |  | 64038 | | 28315 | | 35723 | | 100 | 0 | | 100 | 0 | | 44.2 | 55.8 | |
|  |  | 17/4/17c | 19021 |  | 18779 | | 8142 | | 10637 | | 100 | 0 | | 99.99 | 0.01 | | 43.4 | 56.6 | |
|  |  | 3/5/17 | 23050 |  | 23050 | | 23048 | | 2 | | 100 | 0 | | 100 | 0 | | 99.99 | 0.01 | |
|  |  | 30/5/17b | 35654 |  | 35176 | | 5694 | | 29482 | | 99.4 | 0.6 | | 99.6 | 0.4 | | 17.2 | 82.8 | |
|  |  | 14/6/17b | 20165 |  | 19288 | | 741 | | 18547 | | 100 | 0 | | 96.4 | 3.6 | | 7.4 | 92.6 | |
|  |  | 4/7/17 | 46541 |  | 46160 | | 22970 | | 23190 | | 100 | 0 | | 100 | 0 | | 49.8 | 50.2 | |
|  | lamb | 26/7/16b | 117509 |  | 108013 | | 18957 | | 89056 | | 98.1 | 1.9 | | 97.2 | 2.8 | | 22.3 | 77.7 | |
|  |  | 5/8/16 | 59896 |  | 59282 | | 10578 | | 48704 | | 98.7 | 1.3 | | 99.3 | 0.7 | | 19.8 | 80.2 | |
|  |  | 6/8/16 | 115517 |  | 110047 | | 14776 | | 95271 | | 96.6 | 3.4 | | 92.8 | 7.2 | | 24.0 | 76.0 | |
|  |  | 6/8/16b | 55985 |  | 55742 | | 9183 | | 46559 | | 100 | 0 | | 93.8 | 6.2 | | 22.7 | 77.3 | |
|  |  | 19/8/16 | 17821 |  | 17509 | | 3101 | | 14408 | | 98.9 | 1.1 | | 99.0 | 1.0 | | 19.8 | 80.2 | |
|  |  | 7/9/16b | 6154 |  | 6092 | | 1545 | | 4547 | | 99.0 | 1.0 | | 99.2 | 0.8 | | 27.1 | 72.9 | |
|  |  | 20/9/16e | 15872 |  | 15558 | | 4298 | | 11260 | | 100 | 0 | | 96.8 | 3.2 | | 30.9 | 69.1 | |
|  |  | 17/10/16b | 20394 |  | 20147 | | 7062 | | 13085 | | 99.1 | 0.9 | | 97.7 | 2.3 | | 38.3 | 61.7 | |
|  |  | 24/11/16 | 78712 |  | 78589 | | 26699 | | 51890 | | 98.2 | 1.8 | | 99.9 | 0.1 | | 35.9 | 64.1 | |
|  |  |  |  |  |  | |  | |  | |  |  | |  |  | |  |  | |
|  |  |  |  |  |  | |  | |  | |  |  | |  |  | |  |  | |
|  |  |  |  |  |  | |  | |  | |  |  | |  |  | |  |  | |
|  |  |  |  |  |  | |  | |  | |  |  | |  |  | |  |  | |
|  |  |  |  |  |  | |  | |  | |  |  | |  |  | |  |  | |
|  |  |  |  |  |  | |  | |  | |  |  | |  |  | |  |  | |
|  |  |  |  |  |  | |  | |  | |  |  | |  |  | |  |  | |
|  |  |  |  |  |  | |  | |  | |  |  | |  |  | |  |  | |
|  |  |  |  |  |  | |  | |  | |  |  | |  |  | |  |  | |
|  |  |  |  |  |  | |  | |  | |  |  | |  |  | |  |  | |
|  |  |  |  |  |  | |  | |  | |  |  | |  |  | |  |  | |
|  |  |  |  |  |  | |  | |  | |  |  | |  |  | |  |  | |
|  |  |  |  |  |  | |  | |  | |  |  | |  |  | |  |  | |
| **Farm 3** | ewe | 18/2/16 | 20951 |  | 18607 | | 6456 | | 12151 | | 100 | 0 | | 100 | 0 | | 34.7 | 65.3 | |
|  |  | 18/3/16 | 46011 |  | 41362 | | 5640 | | 41361 | | 100 | 0 | | 0 | 0 | | 14.5 | 85.5 | |
|  |  | 24/5/16 | 20059 |  | 18833 | | 2177 | | 16656 | | 100 | 0 | | 99.9 | 0.1 | | 11.6 | 88.4 | |
|  |  | 3/6/16 | 37484 |  | 36011 | | 10887 | | 25124 | | 100 | 0 | | 100 | 0 | | 30.2 | 69.8 | |
|  |  | 23/6/16 | 3164 |  | 3052 | | 1032 | | 2020 | | 100 | 0 | | 100 | 0 | | 33.8 | 66.2 | |
|  |  | 14/7/16 | 9501 |  | 7274 | | 1384 | | 5890 | | 100 | 0 | | 99.99 | 0.01 | | 19.0 | 81.0 | |
|  |  | 17/8/16 | 14515 |  | 12664 | | 9458 | | 3206 | | 100 | 0 | | 100 | 0 | | 74.7 | 25.3 | |
|  |  | 12/9/16 | 37241 |  | 34121 | | 8613 | | 25508 | | 97.8 | 2.2 | | 99.9 | 0.1 | | 27.6 | 72.4 | |
|  |  | 26/10/16 | 16960 |  | 16020 | | 2572 | | 13448 | | 97.7 | 2.3 | | 94.2 | 5.8 | | 24.2 | 75.8 | |
|  |  | 26/2/17 | 15802 |  | 15026 | | 5061 | | 9965 | | 100 | 0 | | 100 | 0 | | 33.7 | 66.3 | |
|  |  | 20/3/17 | 47909 |  | 47673 | | 26491 | | 21182 | | 100 | 0 | | 86.3 | 13.7 | | 69.3 | 30.7 | |
|  | lamb | 30/6/16 | 73340 |  | 70399 | | 20735 | | 49664 | | 100 | 0 | | 94.0 | 6.0 | | 35.5 | 64.6 | |
|  |  | 14/7/16b | 41540 |  | 38869 | | 5384 | | 33485 | | 97.4 | 2.6 | | 90.8 | 9.2 | | 25.7 | 74.3 | |
|  |  | 2/8/16 | 72649 |  | 71590 | | 8492 | | 63098 | | 98.4 | 1.6 | | 96.2 | 3.8 | | 17.3 | 82.7 | |
|  |  | 17/8/16b | 59106 |  | 58397 | | 17401 | | 40996 | | 100 | 0 | | 96.1 | 3.9 | | 33.7 | 66.3 | |
|  |  | 12/9/16b | 63599 |  | 61649 | | 7437 | | 54212 | | 97.0 | 3.0 | | 91.5 | 8.5 | | 23.6 | 76.4 | |
|  |  | 6/10/16 | 43708 |  | 43384 | | 12411 | | 30973 | | 97.6 | 2.4 | | 99.7 | 0.3 | | 31.4 | 68.6 | |
|  |  | 20/10/16 | 66657 |  | 56366 | | 17395 | | 38971 | | 100 | 0 | | 100 | 0 | | 30.9 | 69.1 | |
|  |  | 14/11/16 | 78869 |  | 77540 | | 23251 | | 54289 | | 96.4 | 3.6 | | 98.9 | 1.1 | | 34.7 | 65.3 | |
|  |  | 8/12/16 | 53727 |  | 48489 | | 3233 | | 45256 | | 100 | 0 | | 96.9 | 3.1 | | 9.8 | 90.2 | |
|  |  |  |  |  |  | |  | |  | |  |  | |  |  | |  |  | |
|  |  |  |  |  |  | |  | |  | |  |  | |  |  | |  |  | |
|  |  |  |  |  |  | |  | |  | |  |  | |  |  | |  |  | |
|  |  |  |  |  |  | |  | |  | |  |  | |  |  | |  |  | |
|  |  |  |  |  |  | |  | |  | |  |  | |  |  | |  |  | |
|  |  |  |  |  |  | |  | |  | |  |  | |  |  | |  |  | |
|  |  |  |  |  |  | |  | |  | |  |  | |  |  | |  |  | |
|  |  |  |  |  |  | |  | |  | |  |  | |  |  | |  |  | |
|  |  |  |  |  |  | |  | |  | |  |  | |  |  | |  |  | |
|  |  |  |  |  |  | |  | |  | |  |  | |  |  | |  |  | |
|  |  |  |  |  |  | |  | |  | |  |  | |  |  | |  |  | |
|  |  |  |  |  |  | |  | |  | |  |  | |  |  | |  |  | |
|  |  |  |  |  |  | |  | |  | |  |  | |  |  | |  |  | |
|  |  |  |  |  |  | |  | |  | |  |  | |  |  | |  |  | |
|  |  |  |  |  |  | |  | |  | |  |  | |  |  | |  |  | |
|  |  |  |  |  |  | |  | |  | |  |  | |  |  | |  |  | |
