# Supplementary material for: Development of amplicon sequencing for the analysis of benzimidazole resistance allele frequencies in field populations of gastrointestinal nematodes
Source: Int J Parasitol Drugs Drug Resist. 2019 Aug 13;10:92–100. doi: 10.1016/j.ijpddr.2019.08.003 (PMC6708983; doi:10.1016/j.ijpddr.2019.08.003)
Supplement: Multimedia component 9 [file mmc9.zip › Supplementary Data S1.docx]

**Schematic representation of the Illumina MiSeq data handling method used to determine the frequencies of isotype 1 β tubulin SNPs in samples of *Teladorsagia circumcincta*.**

Text files containing sequence data (FASTQ files) were generated from the Illumina MiSeq binary raw data outputs, and data analyses were performed using a bespoke pipeline in Mothur v1.39.5 software (Schloss et al., 2009) and Illumina MiSeq standard procedures (Kozich et al., 2013) as described below.

Step 1:

The raw paired-ends reads were analysed to combine the two set of reads for each of our parasite population samples using Mothur v1.39.5 software (make.contigs (file=stability.files, processors=8), which requires ‘stability.files’ as an input. The ‘make.contigs’ command extracts sequence and quality score data from FASTQ files, creating complements of the reverse and forward reads and joins them into contigs. We simply aligned the pairs of sequence reads and compared the alignments to identify any positions where the two reads disagreed.

The output summary of the dataset is shown below.


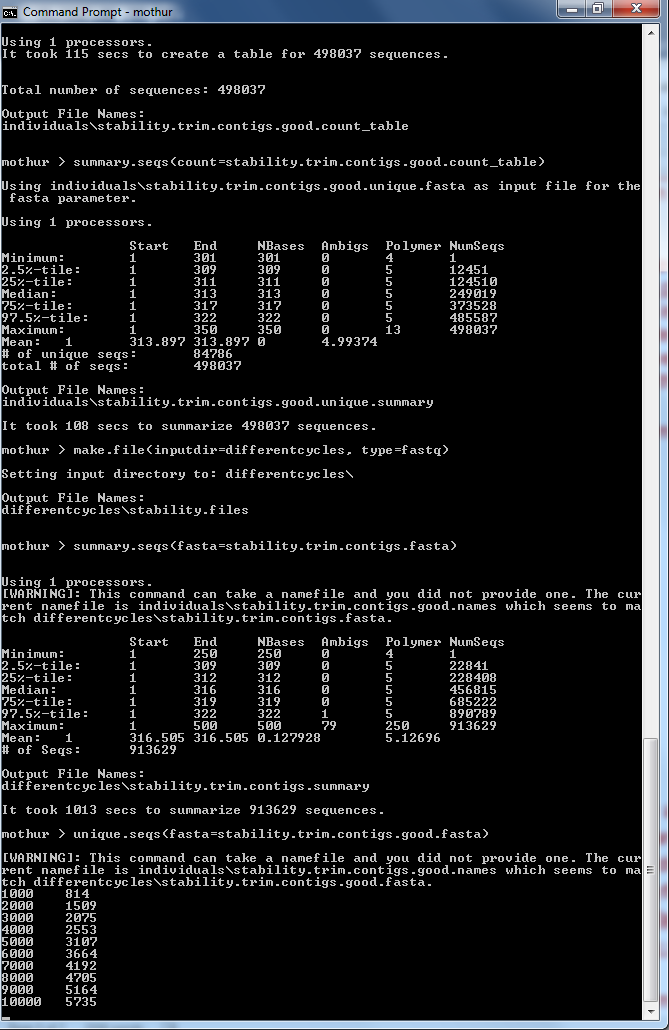


In this example, we have generated 913,629 sequences. The longest read in the dataset is 500 bp (NumSeqs=913,629 - 890,789 =22,840), which is suspicious, because it exceeds the expected length of the target region, hence must be excluded from the dataset. Also, note that at least 2.5% of the sequences have ambiguous base calls (NumSeq=22841). We will take care of this in the ‘screen.seqs’ command below.

Next, we need to remove any sequences with ambiguous bases using the ‘screen.seqs’ command (screen.seqs (fasta=stability.trim.contigs.fasta, maxlength=322, maxambig=0, group=stability.contigs.groups, summary=stability.trim.contigs.summary, processors=8)).

The output summary of the dataset is shown below.


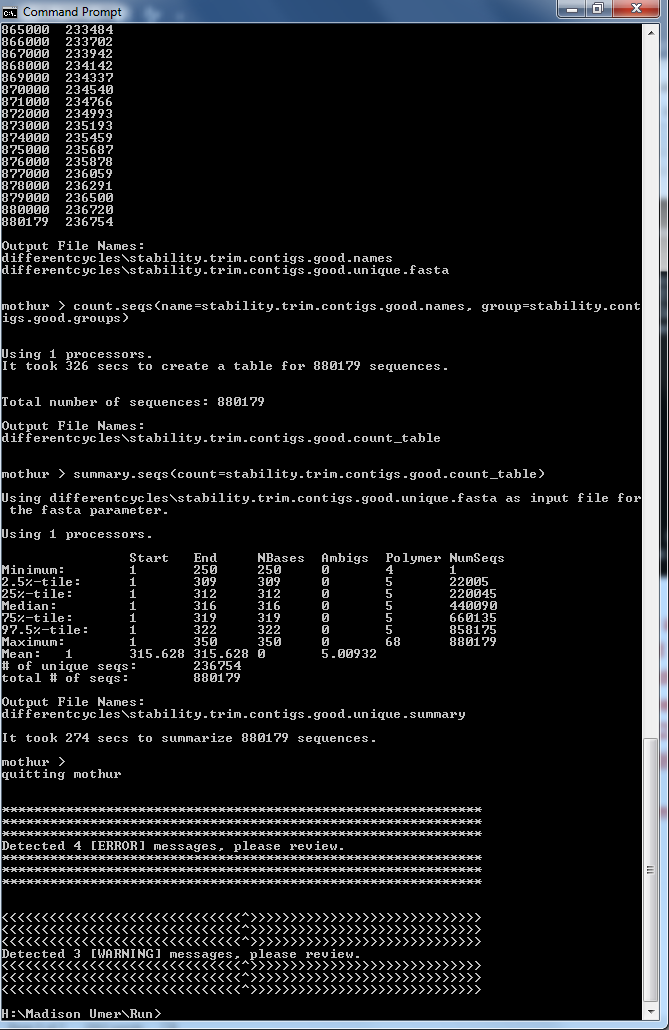


In this example, after removing the ambiguous sequences from the dataset, the above summary tells that we have now total 880,179 out of 913,629 sequences of between 250 and 350 bp filtered.

Step 2:

We can align the above dataset with a *T. circumcincta* reference sequence library of the isotype 1 β-tubulin locus created from the NCBI database (Supplementary Table S2) based on where our sequences start and end corresponding with our primer set (Supplementary Table S1A). We adapt the reference sequence library to be aligned with our *T. circumcincta* dataset using the ‘align.seqs’ command (align.seqs (fasta=stability.trim.contigs.good.unique.fasta, template=consensus_best.pcr.fasta)).

The output summary of the dataset shown below.


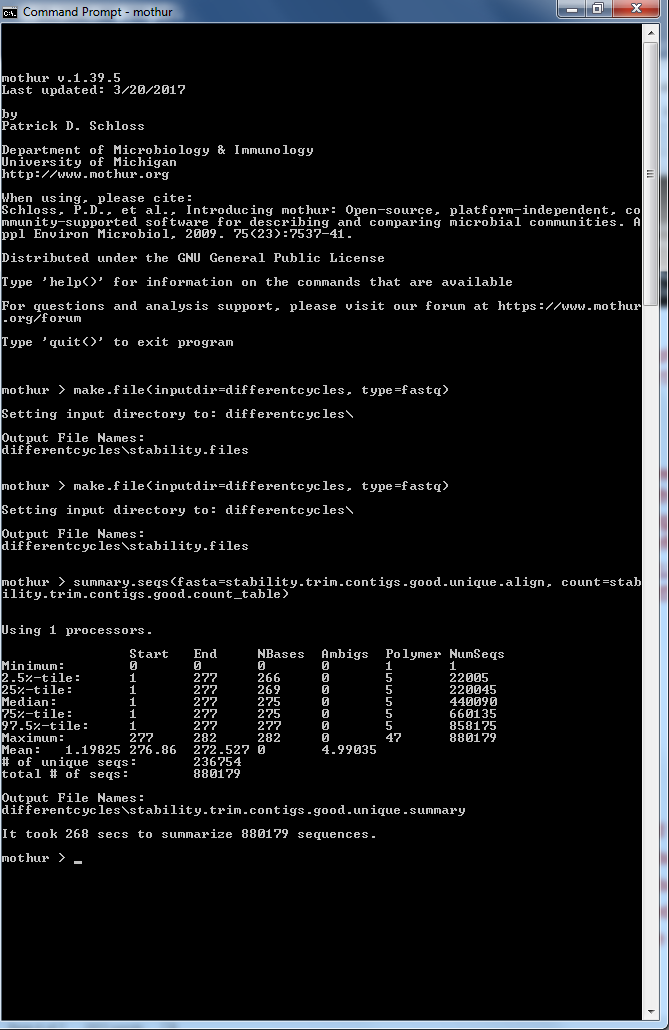


In this example, a total of 880,179 out of the originally generated 913,629 sequences align with the *T. circumcincta* reference sequence library. The nucleotide bases are now all 277 bp, because the *T. circumcincta* reference sequence library is 277 bp in length.

To confirm that these filtered sequences overlap the same region of the *T. circumcincta* reference sequence, we run the ‘screen.seqs’ command to show sequences ending at the 277 bp position (screen.seqs (fasta=stability.trim.contigs.good.unique.align, count=stability.trim.contigs.good.count_table, start=1, end=277, processors=8)).


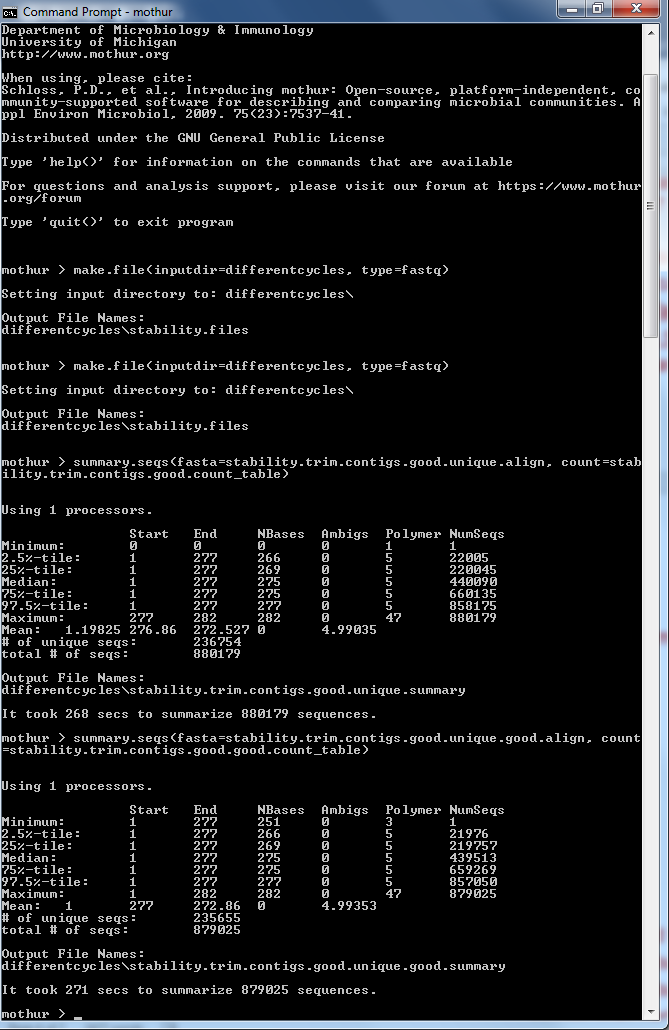
 The output summary is shown below.

This summary shows that we now have a total 879,025 out of 880,179 sequences filtered.

To be validate the accuracy of step 2, we run the ‘classify.seqs’ command to show the taxonomy of the 879,025 sequences, and the sequence detail of each sample (classify.seqs (fasta=stability.trim.contigs.good.unique.good. fasta, count=stability.trim.contigs.good.unique. count_table, template=consensus_sequences.fasta, taxonomy=taxonomy.tax, method=knn, numwanted=3).

Step 3:

Next, we generate a list of consensus sequences for each parasite samples based on the total numbers of filtered sequences (COUNT list) using the ‘unique.seqs’ command (unique.seqs(fasta=stability.trim.contigs.good.unique.good.align, count=stability.trim.contigs.good.good.count_table)).

Example of a COUNT list.


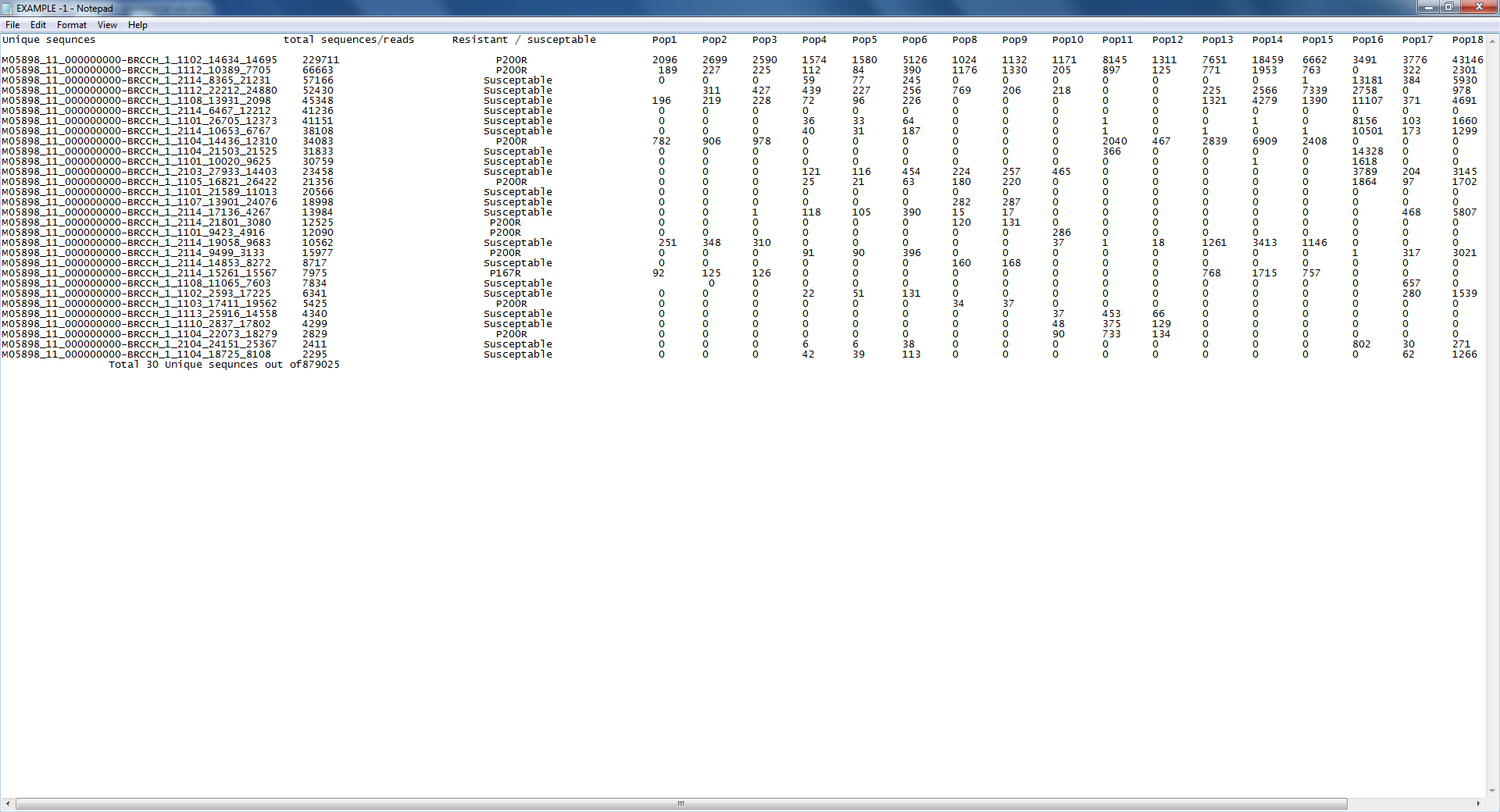


In this example, the COUNT file shows that 30 consensus sequences were generated from 879,025 sequences.

Next we need to confirm that those 30 consensus sequences are *T. circumcincta*. To do this, we generate the FASTA file of the consensus sequences using GENEIOUS v10.2.5 software (Biomatters Ltd, New Zealand).

An example of FASTA files of all samples generated using GENEIOUS v10.2.5 software.


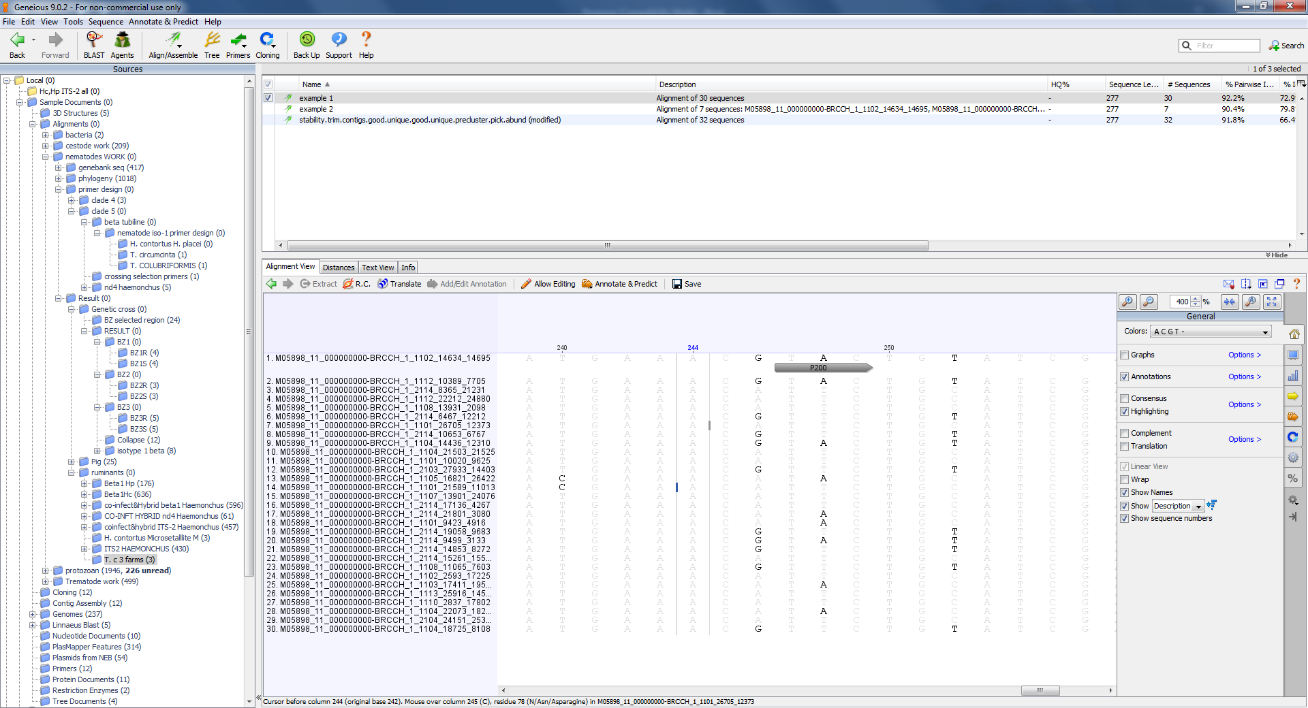

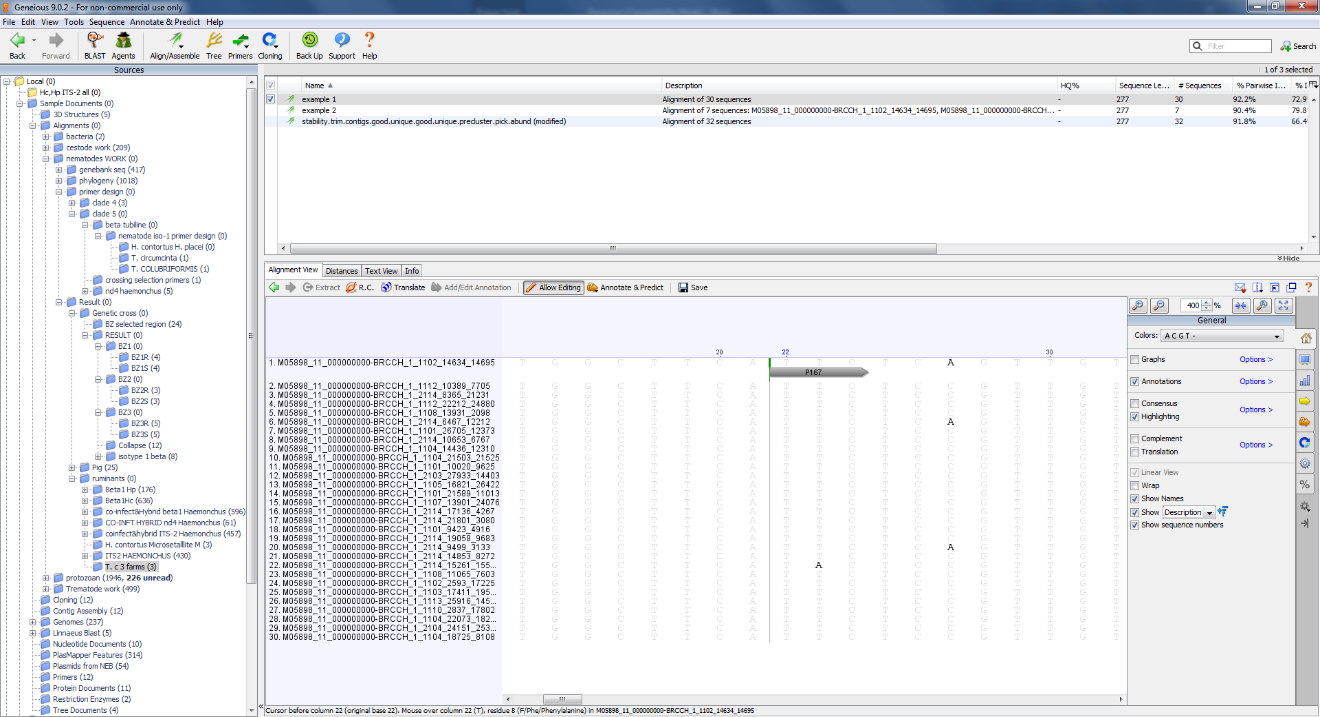


Next we need to determine how many susceptible, P200 and P167 resistant sequences are present in each parasite samples. An example of two samples (pop13 and pop14) is shown below.


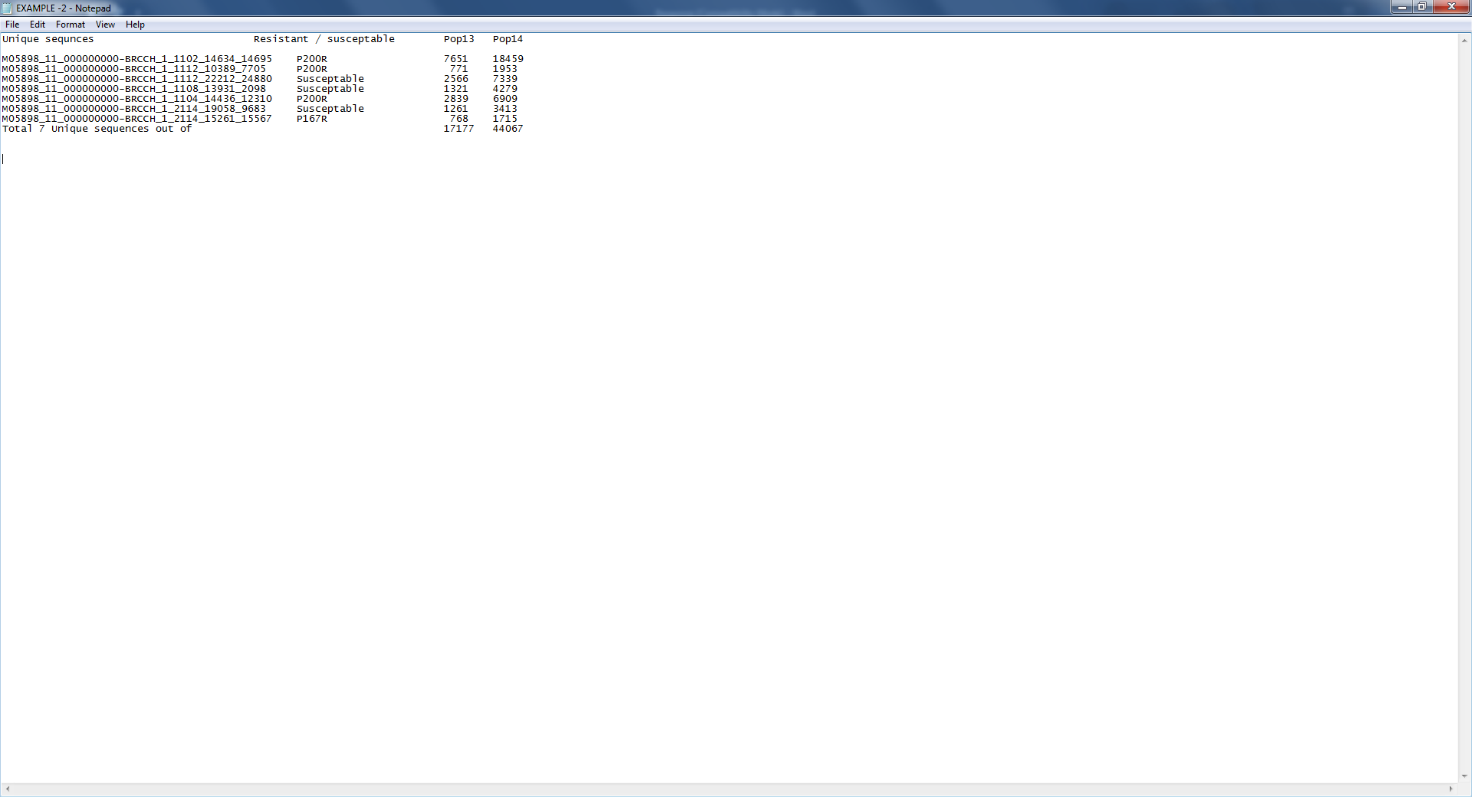
Two samples (pop13 and pop14) COUNT file:

In this example, 7 consensus sequences were generated from 17,177 (pop13) and 44,067 (pop14) sequences. The data also tell us the number of sequence reads present in each consensus sequences (for example M05898_11_000000000-BRCCH_1_1102_14634_14695 has the P200 resistance mutation and is represented by 7,651 (pop13) and 18,459 (pop14) sequences).

Finally, we need to reaffirm that these sequences are *T. circumcincta*. To do this, we run the ‘split.groups’ command to separate the FASTA files of the consensus sequences of each samples (split.groups(fasta=stability.trim.contigs.good.unique.good.unique.precluster.pick.abund.fasta, group=stability.contigs.good.groups). These FASTA files of the consensus sequences are analysed separately on GENEIOUS v10.2.5 software (Biomatters Ltd, New Zealand) to identify susceptible [F200Y (TTC)/ F167Y (TTC)] and resistant alleles [F200Y (TAC)/ F167Y (TAC)].

Example of FASTA file of two samples (pop13 and pop14) in GENEIOUS v10.2.5 software:


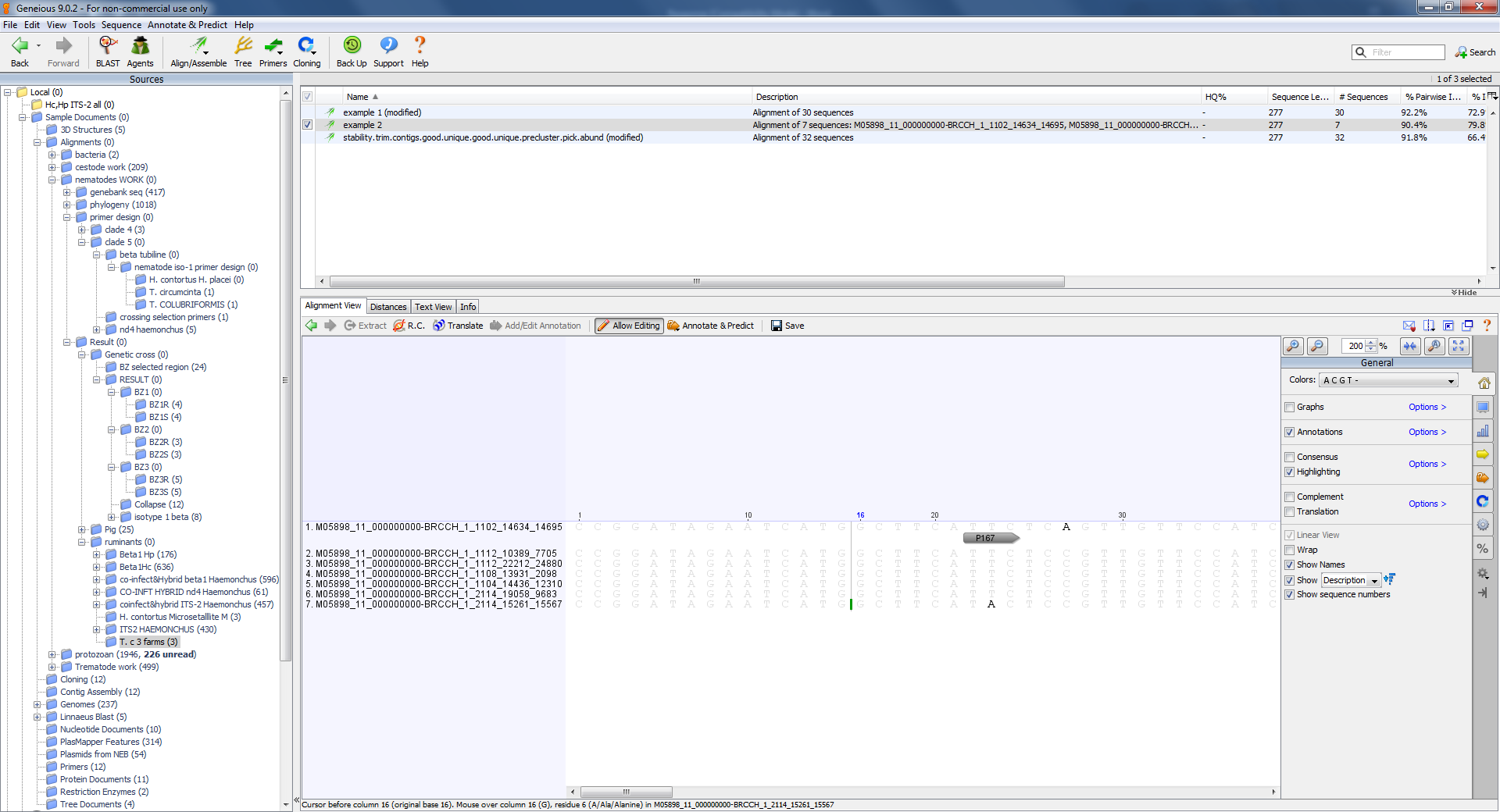

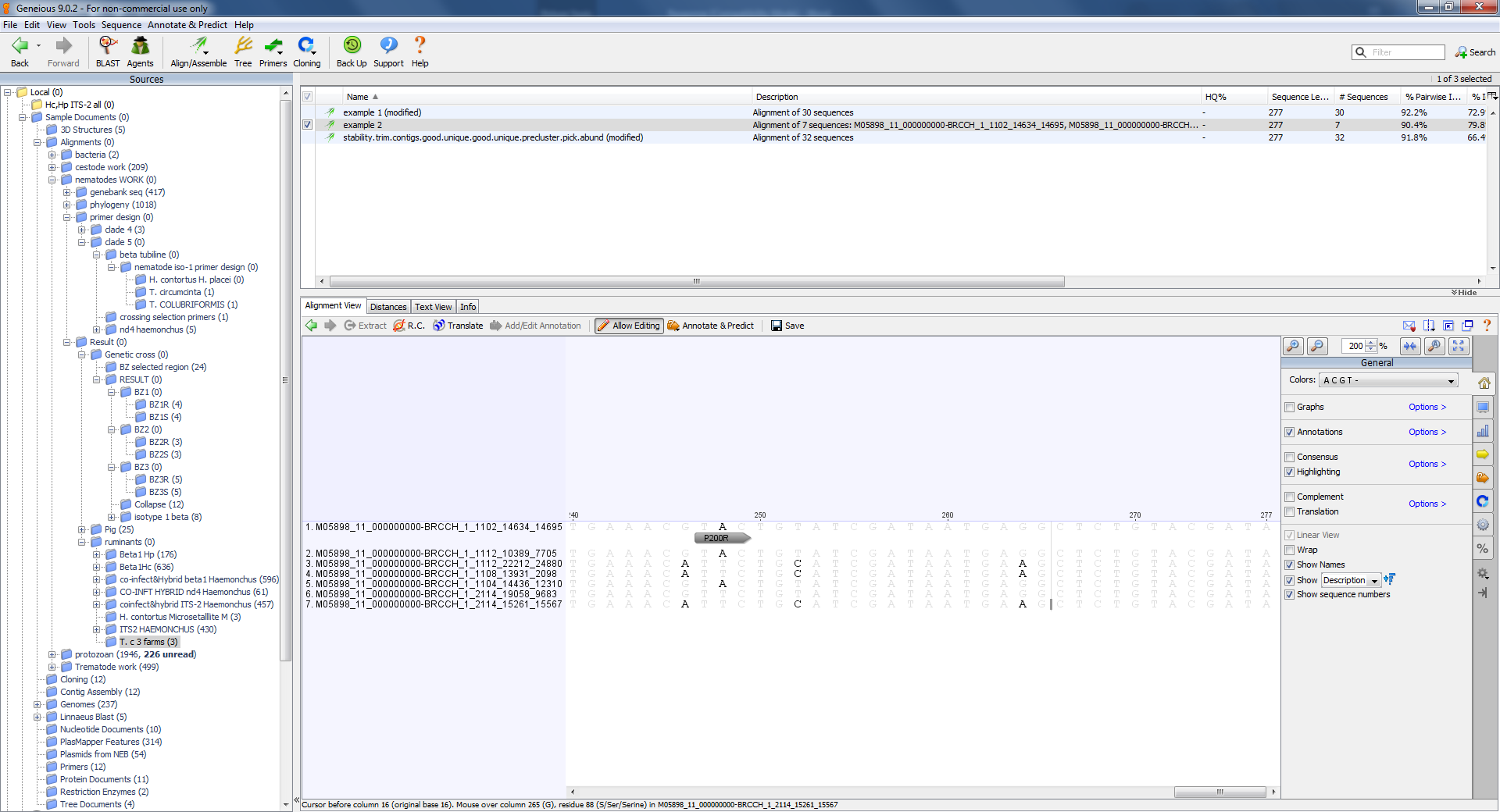


In this example, 7 consensus sequences show the susceptible [F200Y (TTC)/ F167Y (TTC)] and resistant mutations [F200Y (TAC)/ F167Y (TAC)].
